# Coupling of stemness maintenance with cell cycle control in stem cells

**DOI:** 10.3389/fcell.2025.1693489

**Published:** 2025-10-08

**Authors:** Xia Huang, Yujie Wang, Qiushuang Li, Xinyi Li, Congcong Wang

**Affiliations:** College of Life Science and Health, Wuhan University of Science and Technology, Wuhan, Hubei, China

**Keywords:** pluripotent stem cells, Cyclin, cyclin-dependent kinases, Jak1/Stat3 pathway, epigenetic modification, Metabolism, somatic cell reprogramming, adult stem cells

## Abstract

Stem cells are undifferentiated cells characterized by their self-renewal capacity and pluripotency. The multipotent differentiation potential of stem cells grants them significant promise in clinical therapies for tissue injury and organ regeneration. Therefore, the molecular mechanisms underlying the maintenance of stem cell self-renewal and pluripotency have been a major focus of research in the field. In recent years, increasing evidence suggests that cell cycle is not only a central driver of cell division but also participate in controlling stem cell self-renewal and differentiation fate through various pathways. Stem cells, especially embryonic stem cells (ESCs), exhibit unique cell cycle features, with a notably short overall cycle duration, a significantly shortened G1 phase, and a prolonged S phase. This rapid cell cycle not only results in increased cell numbers but is also closely associated with the maintenance of their self-renewal capacity. Pluripotency states (such as naïve, formative, and primed) are tightly linked to specific cell cycle patterns, and this association exhibits species specificity. Elucidating the molecular mechanisms coupling the cell cycle with stemness maintenance is of great significance for the clinical application of stem cells. This review focuses on the cell cycle regulatory network centered around Cyclins and their inhibitors in stem cells, as well as the molecular mechanisms by which core pluripotency factors and cell cycle proteins influence stem cell fate determination. We discuss signaling pathways such as Jak1/Stat3, PI3K/Akt, and Hippo/YAP, and the role of epigenetic regulation, particularly histone modifications, in modulating the expression of differentiation-related and cell cycle-associated genes. Additionally, a brief overview is provided of the unique glycolytic metabolic mode and one-carbon metabolism in stem cells, along with their relationship with epigenetic modifications and rapid proliferative characteristics. Moreover, we analyze the regulatory functions of cell cycle regulators such as Cyclins and checkpoint protein p53 in somatic cell reprogramming and the fate determination of adult stem cells including neural and hematopoietic stem cells (HSCs). Practical strategies based on cell cycle regulation are discussed, along with prospects and challenges for their applications in regenerative medicine.

## 1 Introduction

Embryonic stem cells (ESCs), primarily derived from the inner cell mass of blastocysts, are a population of undifferentiated cells with the capacity to unlimited proliferation, self-renewal, and maintenance of pluripotency (Evans and Kaufman, 1981; Martin, 1981). Pluripotency refers to the ability to differentiate into the three germ layers: ectoderm, mesoderm, and endoderm (Lu et al., 2001). Previous studies have shown that ESC pluripotency is regulated by core pluripotency factors such as Sox2, Oct4, and Nanog, which maintain the undifferentiated state of ESCs, and suppress the expression of differentiation genes (Boyer et al., 2005; Hall et al., 2009; Mitsui et al., 2003; Surani et al., 2007). Since the establishment of the first mouse ESC (mESC) line, significant breakthroughs have been achieved in ESC-related research (Evans and Kaufman, 1981; Martin, 1981). More recently, the mechanisms by which ESCs couple cell cycle regulation to maintain pluripotency have been progressively elucidated (Chaigne et al., 2020).

Many studies in the ESC field have confirmed that the pluripotent state of ESCs is associated with a specific cell cycle profile (Coronado et al., 2013; Pauklin and Vallier, 2013). The cell cycle is divided into four phases: G1 (pre-DNA synthesis), S (DNA synthesis), G2 (post-DNA synthesis), and M (mitosis). Its progression is primarily regulated by a set of proteins centered on Cyclins, including Cyclin-dependent kinases (CDKs) and Cyclin-dependent kinase inhibitors (CKIs). The cell cycle relies on two peaks of gene expression: during the G1/S transition and the G2/M transition. During G1, growth factors induce the expression of CyclinD (D1, D2, or D3), which binds to CDK4/6 to form active complexes, initiating the sequential phosphorylation of the retinoblastoma protein (RB) (Hydbring et al., 2016). This process partially relieves RB’s inhibition of E2F transcription factors, promoting the expression of key G1/S transition genes such as *CyclinE* (Liu et al., 2019). After the restriction point, the CyclinE-CDK2 complex further hyperphosphorylates RB *via* positive feedback, fully activating E2F and phosphorylating replication initiation factors such as CDC6 to facilitate pre-replication complex assembly, preparing for DNA synthesis (Narasimha et al., 2014). Upon entry into S phase, the Cyclin A-CDK2 complex supersedes Cyclin E-CDK2, ensuring the timely initiation and progression of DNA replication (Coverley et al., 2002). In G2 phase, CyclinA levels decline, and the activity of CyclinB-CDK1 is regulated by CDC25 phosphatases, forming a molecular switch that governs the transition from G2 to M phase. During the G2/M transition, CDK1 binds to CyclinA or B, with these complexes being essential for proper mitotic entry (Errico et al., 2010).

Cyclins and CDKs, as the primary executors of cell cycle regulation, are negatively modulated by two major CKI families: INK4 and Cip/Kip. The INK4 family mainly includes p16, p15, p18, and p19, which selectively inhibit the CDK4/CDK6 kinase complexes in the G1 phase by directly binding to CDK4/6 and preventing their association with and activation by D-type Cyclins, thereby maintaining the Rb in a hypo-phosphorylated, active state (Serrano et al., 1993; Sherr and Roberts, 1999). The Cip/Kip family includes p21, p27, and p57, which have a broader inhibitory spectrum, capable of suppressing multiple Cyclin-CDK complexes in G1 and S phases, and exhibit a dual regulatory role on the assembly of Cyclin-CDK complexes at different concentrations, either promoting or inhibiting their formation (Besson et al., 2008; Sherr and Roberts, 1999). In naïve-state ESCs, the expression levels of Cip/Kip family members (especially p21) are typically low, which helps sustain high CDK2 activity and ensure rapid passage through G1, thereby supporting self-renewal and rapid proliferation (Neganova and Lako, 2008). Expression of *p57* is essential for genomic imprinting and normal development (Matsumoto et al., 2011). As ESCs initiate differentiation, expression of *p21* and *p27* is markedly upregulated. At this stage, they inhibit the activity of the Cyclin E-CDK2 complex, forcing cells out of the high-proliferation cycle, lengthening G1, and promoting differentiation (DeVeale et al., 2022). p57 is a core molecule for maintaining the quiescent state of hematopoietic stem cells (HSCs) and neural stem cells (NSCs) (Furutachi et al., 2013; Zou et al., 2011). In quiescent stem cells, p27 protein levels are high; by inhibiting the Cyclin E-CDK2 complex, they effectively lock cells in G0/G1 to prevent excessive proliferation and attrition. Upon receiving activating signals, p27 is degraded *via* phosphorylation and ubiquitination pathways, relieving inhibition on CDK2 and allowing cells to re-enter the proliferative cycle (Besson et al., 2006; Doetsch et al., 2002). *p16* plays a dominant role in reinforcing quiescence and aging-related proliferative blockades. In young adult stem cells (ASCs), *p16* expression is relatively low; however, with aging or persistent stress, its expression rises significantly (Molofsky et al., 2006). High levels of p16 inhibit CDK4/6, augment RB-mediated cell-cycle arrest, and lock stem cells into a deeper, more irreversible quiescent state, potentially leading to senescence (D'Arcangelo et al., 2017). In ASCs such as HSCs and NSCs, high levels of CKIs constitute a key mechanism for actively maintaining quiescence, functioning as reversible “molecular brakes” that keep stem cells in a standby state, with INK4 family members expressed at comparatively lower levels. When stem cells are activated and enter division, if their progeny decide to proceed toward terminal differentiation, CKIs will be upregulated again, leading to an irreversible exit from the cell cycle and promotion of differentiation.

Some scholars believe that because stem cells exhibit high expression of lineage-specific genes during G1, the prolongation of G1 phase makes them more susceptible to differentiation signals, thus G1 is considered a “sensitive period” for differentiation (Chaigne et al., 2020; Coronado et al., 2013; Dalton, 2013; Dalton, 2015; Pauklin and Vallier, 2013; Pauklin and Vallier, 2014; Singh et al., 2014). Compared with somatic cells, ESCs demonstrate rapid proliferation, with a markedly shortened cell cycle, predominantly characterized by a significantly reduced G1 phase. In typical ESC populations, S phase cells can account for 60%–70%, while G1 phase cells occupy only 15%–20%, a stark contrast to the G1-dominant cycle of somatic cells (Ter Huurne and Stunnenberg, 2021). Research indicates that the abbreviated G1 phase in ESCs is regulated in part by the interplay between the classical oncogenes *MEK1/2* and the tumor suppressor gene *TP53*, which collectively drive the G1/S transition (Jiang et al., 2022). This distinctive cell cycle pattern is considered a fundamental basis for maintaining the undifferentiated state of ESCs. At the same time, this pattern also ensures rapid cell proliferation during early embryonic development (White and Dalton, 2005). During mammalian embryogenesis, pluripotent cells initially undergo rapid division phases from the preimplantation to the early postimplantation stages (Stead et al., 2002). This distinctive mode of cell division effectively promotes the rapid expansion of pluripotent stem cell (PSC) populations before gastrulation. This conserved regulation of the cell cycle has been confirmed across various model organisms, including fruit flies (Edgar and Lehner, 1996), zebrafish (Yarden and Geiger, 1996), and African clawed frogs (Murray and Kirschner, 1989), in the context of unipotent cells transitioning to a differentiated state, with marked changes in proliferation rates. These findings thoroughly demonstrate that the mechanisms governing cell cycle regulation are highly conserved evolutionarily and play a core role in determining cell fate and maintaining cell characteristics (Boward et al., 2016; Liu et al., 2019).

PSCs represent a heterogeneous population comprising a spectrum of functional states. These states are characterized by distinct developmental potentials, metabolic profiles, epigenetic configurations, and signaling pathway dependencies, and are broadly classified into naïve, formative, and primed pluripotency states (Table 1). A deep understanding of the cell cycle characteristics of these states is crucial for elucidating mechanisms of pluripotency maintenance and exit. Cell cycle dynamics are not only a passive readout of these states but also an active regulator of their maintenance and transitions. Notably, the key properties of these states show important differences between mouse and human cells.

**TABLE 1:** Characteristics of ESCs in different states.

Feature	Naïve state	Formative state	Primed state
Developmental Stage	ICM of the blastocyst	Peri-implantation transition	Post-implantation Epiblast (Epi)
Pluripotency	Broadest (includes embryonic and extra-embryonic potential)	Transitional, competent for germline entry	Restricted (biased towards specific lineages)
Colony Morphology	Dome-shaped, with indistinct cell boundaries	Intermediate	Flat, monolayer with epithelial-like appearance
Signaling Pathways	LIF/Stat3 Wnt/β-catenin	FGF/Erk	FGF/Erk TGF-β/Activin/Nodal
Cell Cycle	Short cycle, abbreviated G1 phase, rapid proliferation	Lengthening cycle	Extended cycle, stringent G1/S checkpoint
Epigenetic Features	X-Chromosome: Both X chromosomes active (in females) DNA: Global hypomethylation Chromatin: Open, highly plastic	X-Chromosome: X-chromosome inactivation (XCI) initiated DNA: Methylation levels rising Chromatin: Undergoing restructuring and closing	X-Chromosome: One X chromosome silenced (in females) DNA: Global hypermethylation Chromatin: Closed, relatively stable

Naïve pluripotency corresponds to the pre-implantation ICM or the epiblast of the mouse embryo. This state exhibits the broadest developmental potential, enabling differentiation into all embryonic and extraembryonic lineages and facilitating efficient chimera formation. A hallmark of naïve pluripotent cells is their distinct cell cycle structure, characterized by a shortened G1 phase and an extended S phase, resulting in a total cell cycle length of approximately 12–14 h. This unique cycle architecture is driven by high activity of CDK2, Cyclin A, and Cyclin B, accompanied by silenced expression of G1-phase CDK inhibitors such as p21 and p27, resulting in a hyperphosphorylated, inactive Rb protein. This configuration is considered favorable for rapid proliferation and minimizes the time for external differentiation signals to influence cells in G1, thereby passively maintaining pluripotency. Naïve-state cells rely on glycolytic metabolism to supply biosynthetic precursors while maintaining low reactive oxygen species (ROS) levels, aligning with their short cell cycle. Epigenetically, Naïve cells exhibit global DNA hypomethylation and abundant H3K27me3 marks to suppress differentiation programs, with X chromosome reactivation observed in female cells. Maintenance of this state strictly depends on dual inhibition of the LIF/Stat3 pathway and the GSK3β/MEK/Erk pathways (“2i” conditions) (Coronado et al., 2013; Weinberger et al., 2016).

Primed pluripotency is characteristic of the post-implantation mouse epiblast. This state exhibits restricted developmental potential, lacking the ability to differentiate into the trophoblast lineage and displaying increased responsiveness to differentiation signals. Unlike naïve pluripotency, primed pluripotent cells undergo a substantially prolonged cell cycle, often lasting more than 24 h, primarily attributable to an elongated G1 phase. The upregulation of G1-phase CDK inhibitors, diminished CDK2 activity, and reduced Rb phosphorylation collectively reinforce the G1 checkpoint and decelerate cell cycle progression. This extended G1 phase offers a crucial temporal window for the integration of extracellular cues and the initiation of lineage-specific transcriptional programs. Metabolically, primed cells shift from glycolysis toward oxidative phosphorylation (OXPHOS) to generate more ATP to support cellular activities. Epigenetically, DNA methylation is increased, and chromatin adopts a more closed and compact state, with X chromosome inactivation in female cells. Maintenance of the primed state largely depends on activation of the FGF2 and Activin A/Nodal signaling pathways (Nichols and Smith, 2009; Singh et al., 2015).

Formative pluripotency represents a recently defined intermediate state situated between naïve and primed pluripotency, corresponding to the epiblast at implantation onset. Cells in this state have lost key naïve pluripotency features but have not yet fully acquired primed characteristics. Regarded as a transitional phase, formative pluripotency enables cells to respond efficiently to inductive signals. Evidence suggests that these cells display an intermediate cell cycle structure, marked by a significantly lengthened G1 phase, yet the total cycle duration remains shorter than that of primed cells—reflecting their dynamic and metastable nature. Concurrently, their signaling dependencies and epigenetic landscape undergo rapid remodeling in preparation for lineage specification (Smith, 2017).

Notably, interspecies differences are key to understanding pluripotent states. Human PSCs (hPSC) sunder standard culture conditions (using FGF2 and Activin A) are typically in a primed state rather than the naïve state observed in mouse. Even under optimized “naïve” culture conditions, the G1 phase of hPSCs is generally longer than that of mouse naïve ESCs, and their cell cycle architecture more closely resembles mouse primed or formative states. Additionally, in human cells, the p53 and p16/Rb pathways exert a stronger barrier to reprogramming and maintenance of the naïve state than in the mouse, directly affecting cell cycle dynamics and proliferative potential (Table 2). Consequently, findings from mESC research do not fully translate to human systems (Becker et al., 2010; Theunissen et al., 2014).

**TABLE 2:** Comparison of characteristics between mouse and human ESCs.

Feature	mESCs	hESCs
Canonical State	Naïve. mESCs are directly derived from and stably maintained in the naïve state of pluripotency.	Primed. Conventional hESCs (and hiPSCs) naturally reside in the primed state of pluripotency.
Signaling Pathways	LIF/Stat3 signaling is crucial for self-renewal. Inhibition of BMP and Erk/MAPK pathways helps maintain the naïve state.	FGF2 (bFGF) and Activin A/TGF-β signaling are essential for self-renewal. Wnt signaling also plays a supportive role.
Colony Morphology	Form smooth, compact, and dome-shaped colonies with indistinct cell boundaries.	Form flat, loose colonies with more clearly defined cell boundaries.
X-Chromosome Status (Female)	XaXa (Both active): Naïve female mESCs have two active X chromosomes; X-inactivation has not occurred.	XaXi (One inactive): Primed female hESCs have one inactivated X chromosome. This can be reversed upon reprogramming to a naïve state.
Metabolic Profile	Rely more heavily on glycolysis for energy production, even in the presence of oxygen (aerobic glycolysis/Warburg effect).	Rely more on OXPHOS for efficient ATP generation.
Epigenetic Landscape	More open: Characterized by globally low DNA methylation and a more open chromatin configuration. This makes them more amenable to genetic manipulation and reprogramming.	More closed: Feature higher global DNA methylation and a more restrictive chromatin structure. This confers greater stability but reduced plasticity.

Pluripotent states of stem cells are not static endpoints. Rather, they are a “quasi-steady state” that is dynamically shaped by the cell cycle, and supported by energy metabolism and the epigenetic landscape in a finely balanced state. Their unique rapid proliferation cycle, especially the shortened G1 phase, serves both as the engine that maintains pluripotency and as a source of genomic instability risk. Therefore, one goal of the pluripotency network is to build a coupled regulatory system that integrates the cell cycle, metabolism, epigenetics, and signaling pathways to achieve self-renewal, fate determination, and genomic safeguarding amid rapid proliferation. This article systematically articulates the coupled mechanism between the cell cycle and stemness maintenance in stem cells, analyzes how the cell cycle regulatory network—centered on Cyclins and CDKs—regulates stem cell pluripotency maintenance, and provides a comprehensive discussion of signaling pathways such as LIF/Stat3, epigenetic regulation like histone modifications, metabolism, and somatic cell reprogramming as regulators of the cell cycle.

## 2 Specific mode of cell cycle control by Cyclins in ESCs

### 2.1 Crosstalk of cell cycle regulators and pluripotent factors in ESCs

**FIGURE 1:**
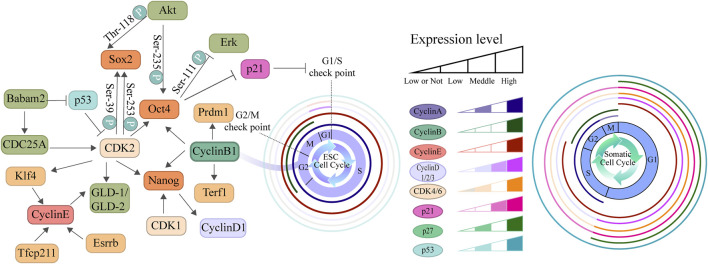
Comparative diagram of cell cycle regulation in ESCs and somatic cells. This diagram illustrates the differences in cell cycle structure and the expression patterns of regulatory factors between ESCs and somatic cell. The left panel depicts the core cell cycle regulatory network centered around cyclins, CKIs, and pluripotency factors. The two cell cycle diagrams on the right represent the typical cell cycle structures of ESCs and somatic cells, respectively, highlighting the significant differences in the duration of the G1, S, G2, and M phases between the two cell types. The relative expression levels of key cell cycle regulatory factors across these phases are indicated by color gradients within concentric rings. The results demonstrate that the enhanced expression of Cyclin-CDK complexes in ESCs, coupled with their abbreviated G1 phase and prolonged S phase, collectively contribute to the maintenance of their pluripotent state.

The distinctive cell cycle architecture observed in ESCs results from the synergistic effects of multiple regulatory layers, including transcription factors, epigenetic modifications, signaling pathways, metabolism, and cell cycle regulators (Nardiello et al., 2011). Studies indicate that, unlike highly differentiated cells, ESCs maintain expression levels of cell cycle genes such as *CyclinE-CDK2* and *CyclinA-CDK2* throughout the cell cycle, with *CyclinE-CDK2* playing a crucial role in the G1/S transition, whereas *CyclinA-CDK2* and *CyclinB-CDK1* drive rapid progression through S phase and G2/M phase, respectively (Figure 1) (Liu et al., 2019). Furthermore, the expression levels of CDK inhibitors are relatively low in ESCs, further relieving the inhibition of CDK activity (Calder et al., 2013; Egozi et al., 2007; Zhu H. et al., 2014). Concurrently, the highly phosphorylated state of RB, leading to a continuous release of its suppression of E2F transcription factors, enabling rapid passage through the G1/S checkpoint (Koledova et al., 2010; Krishnan et al., 2022).

In recent years, numerous studies have reported the associations between cell cycle regulators and pluripotency control. Knockdown of individual CyclinD isoforms (D1, D2, D3) in human ESCs (hESCs) allows for self-renewal but results in the upregulation of lineage markers, including mesodermal and endodermal genes. Double knockdown of CyclinD promotes spontaneous differentiation toward the endodermal lineage but reduces the capacity for ectodermal differentiation. When all CyclinD isoforms are suppressed, hESCs lose their self-renewal ability and differentiate predominantly into the endoderm (Pauklin and Vallier, 2014). Gonzales et al. found that prolonging the G2 phase specifically upregulates *CyclinB1* expression, suggesting that *CyclinB1* may be a key factor in main-taining pluripotency during G2 phase. Knockdown of CyclinB1 in hESCs results in a sharp decrease in the expression of pluripotency markers such as Oct4, Nanog, Terf1, and Prdm1, directly confirming its close association with pluripotency. Conversely, overexpression of *CyclinB1* can enhance the expression of these markers. These findings collectively indicate that *CyclinB1* serves as a central hub connecting G2 phase regulation to the maintenance of pluripotency (Gonzales et al., 2015). Downregulation of CDK2 also reduces the expression of pluripotent factors (Oct4, Sox2, Nanog, Klf4), induces the expression of three germ layer differentiation markers (Cdx2, Pax6, Nestin, Fgr5, Brachyur, Afp), and triggers lineage commitment (Neganova et al., 2009; Spelat et al., 2012). Additional research suggests that Cyclin-CDK complexes prevent ubiquitin-mediated degradation of Sox2, Oct4, and Nanog by promoting their interaction with membrane proteins (Savatier et al., 1994). CDK2-mediated phosphorylation of Sox2 at Ser39 and Ser253 is critical for maintaining its activity (Bar-On et al., 2010). These studies demonstrate that cell cycle mechanisms play a central role in regulating stem cell pluripotency and self-renewal. Similarly, core pluripotency factors are directly involved in cell cycle regulation. To illustrate, Akt-mediated phosphorylation of Sox2 at Thr-118 contributes to the maintenance of the pluripotent state (Jeong et al., 2010). Erk-mediated phosphorylation of Oct4 at Ser-111 promotes its ubiquitination and subsequent degradation (Spelat et al., 2012). Conversely, Akt-mediated phosphorylation of Oct4 at Ser-235 stabilizes the protein and facilitates its interaction with Sox2, thereby supporting stem cell self-renewal (Lin Y. J. et al., 2012). *Oct4* can promote the G1/S transition by downregulating *p21* expression (Lu Y. et al., 2019), while *Nanog* is capable of upregulating *CyclinD1* expression (Han et al., 2012). The initial pluripotency network transcription factors, including Klf4 and Esrrb, participate in the transcriptional regulation of CyclinE (Gonnot et al., 2019). This bidirectional regulation ensures that ESCs can proliferate rapidly while maintaining their undifferentiated state.

Recent studies have revealed that Cyclins and CDKs actively participate in regulating the pluripotency network through various pathways. CDK1 modulates H3K79me2 methylation by phosphorylating Dot1L, thereby influencing the differentiation tendency of ESCs toward the endoderm (Michowski et al., 2020). Simultaneously, the inhibition of CDK1 activity can activate the p53-Noxa-Mcl1 axis, leading to selective apoptosis of ESCs and further affecting the expression of *Oct4* and *Nanog*, ultimately resulting in stem cell differentiation (Huskey et al., 2015). Additionally, in germline stem cells, *CyclinB3* sustains self-renewal through bam-dependent mechanisms, while *CyclinE/CDK2* promotes germ cell proliferation *via* the Gld-1/Gld-2 pathway (Chen D. S. et al., 2018; Fox et al., 2011). These findings suggest that cell cycle proteins exert multi-layered regulatory roles in cell fate determination.

During embryonic germ layer differentiation, the functions of cell cycle proteins are equally crucial. The loss of G1 phase Cyclins weakens the stability of pluripotency factors in ESCs, making them more prone to differentiate into the trophectoderm (TE) lineage (Liu L. J. et al., 2017). Meanwhile, CyclinD promotes neural ectoderm differentiation by binding to regulatory regions of developmental genes and inhibits endodermal gene expression (Pauklin et al., 2016; Pauklin and Vallier, 2013). Furthermore, decreased expression of *CDK4* reduces phosphorylation of the Smad-Stat3 axis, favoring the differentiation of mesenchymal stem cells (MSCs) into neuroprogenitors (Kim et al., 2016; Pauklin and Vallier, 2013). Notably, in hESCs, differentiation tendencies vary across different phases of the cell cycle. For instance, the early G1 phase favors endodermal differentiation, whereas the late G1 phase promotes neuroectodermal fate. Additionally, the CyclinD-CDK4/6 complex regulates Activin/Nodal signaling through phosphorylation of Smad2/3, further influencing endodermal differentiation (Pauklin and Vallier, 2013).

Certain cell cycle regulatory complexes are closely associated with the maintenance of stem cell pluripotency. DREAM complex proteins were first discovered in *C. elegans*, where they primarily maintain normal cellular function by regulating the expression of genes involved in cell proliferation (Mages et al., 2017). DREAM is widely present across multiple species and spans all stages of organismal growth and development; aberrant expression is often closely linked to tumorigenesis. In mammals, the DREAM complex is mainly composed of p107/p130, E2F4/5, DP, and MuvB (Sadasivam and DeCaprio, 2013). This complex is highly conserved evolutionarily. DREM and dREAM complexes in *C. elegans* and *Drosophila* are homologs of the DREAM complex in mammals (Schade et al., 2019). The MuvB complex, a key regulator during mitosis, comprises Lin9, Lin37, Lin52, Lin54, and Rbbp4, with Lin54 and Lin52 maintaining the characteristic extended S-phase of ESCs by regulating CyclinB1/CDK1 (Litovchick et al., 2007; Wang C. C. et al., 2022). Notably, the MuvB complex undergoes dynamic remodeling during the cell cycle: at the G1/S transition, it dissociates from the repressive DREAM (*Drosophila*, RB, E2F, and Myb) complex and associates with B-Myb to form the Myb-MuvB complex, which activates G2/M phase gene expression and suppresses mesoderm/endoderm differentiation markers, thus coupling the cell cycle process with pluripotency maintenance (Guiley et al., 2015; Litovchick et al., 2007). Rbbp4 is not only a major component of the MuvB complex but also serves as an important histone chaperone, playing a key role in maintaining pluripotency in mESCs. Rbbp4 specifically binds to endogenous retrotransposon (TE) elements; on the one hand, it recruits G9a to deposit H3K9me2 modifications at ERVL-class transposons, and on the other hand, it recruits Kap1 to establish H3K9me3 marks at ERVK-class elements. Meanwhile, it cooperates with the chromatin remodeling factor CHD4 to maintain nucleosome occupancy density in heterochromatic regions, collectively repressing the transcriptional activity of totipotency-related genes and transposons. When Rbbp4 function is lost, this heterochromatin barrier is dismantled, leading to aberrant activation of TEs, which drives mESCs to reprogram into totipotent 2C-like cells (2CLCs), accompanied by typical 2CLC phenotypes such as delayed proliferation, increased apoptosis, and G1/S phase arrest. Meanwhile, the repression of key genes in the trophoblast lineage is alleviated, significantly enhancing the potential for differentiation into the outer trophoblast lineage (Ping et al., 2023). The dynamic activation and repression of DREAM subunits directly regulate the activity of cell-cycle proteins. In the G0 quiescent state, p107/p130, DP, E2F, and MuvB interact to suppress the expression of cell-cycle genes (Uxa et al., 2019). After cells receive extracellular growth factor signals and pass the restriction (R) point—the classical checkpoint marking the transition from G1 to S—the expression of genes required for DNA synthesis is activated, the RB-mediated repression of E2F transcription factors is relieved, and Cyclin E and Cyclin D, together with CDKs, can mitigate RB-mediated E2F inhibition; activator E2Fs (E2F1, E2F2, and E2F3) contribute to the expression of early cell-cycle genes during the G1/S transition (Litovchick et al., 2007). Subsequently, MuvB, through interactions with B-myb and FoxM1, induces the expression of late cell-cycle genes. It is the periodic activation and repression of these complex members that ensures the orderly expression of downstream cell-cycle proteins (Fischer and Müller, 2017; Kelleher and O'Sullivan, 2016; Liu et al., 2019; Sadasivam et al., 2012).

Some researchers propose that E2F2 directly binds the B-Myb promoter to promote its transcription. B-Myb, by binding the E2F2 promoter to enhance its expression, together with E2F2, co-activates FoxM1, forming the E2F2/B-Myb/FoxM1 core regulatory network that drives cell-cycle progression (Werwein et al., 2020). E2F has been demonstrated to be a principal target of the tumor suppressor RB (Roufayel et al., 2021). E2F proteins bind RB, and their transcriptional activity is repressed in G0 and early G1 (Inoshita et al., 1999). The RB–E2F complex represses transcription of these genes, an repression relieved by RB phosphorylation and the concomitant release of E2F transcription factors, thereby enabling the expression of factors regulating the cell cycle (Engeland, 2022; Kato et al., 1993). In pluripotent cells, complexes of CDK2 with Cyclin A/E continuously phosphorylate RB family proteins, resulting in sustained release of E2F transcription factors and activation of downstream target genes. On one hand, the persistently activated E2F targets drive pluripotent cells past the G1 checkpoint, directly contributing to a shortened G1 phase; on the other hand, E2F–driven activation of the Cyclin A/E–CDK2 complex creates a positive feedback loop that further phosphorylates RB, reinforces E2F activity, and ensures the irreversibility of the G1/S transition (Stead et al., 2002). Precise regulation of E2F activity is crucial for maintaining cellular pluripotency: when RB is activated, its E2F-binding domain inhibits E2F transcription factor activity, leading to an H3K27me3-enriched repressive chromatin environment at the promoters of pluripotency genes, thereby hindering establishment and maintenance of pluripotency; conversely, excessive E2F activity can trigger genomic stress responses, increase apoptosis, and disrupt chromatin modifications, thereby hindering the generation of high-quality PSCs. Experiments show that pluripotency can be effectively promoted only when E2F activity is moderately activated, by relieving transcriptional repression of pluripotency genes while avoiding cellular homeostasis imbalance (Kareta et al., 2015).

Although extensive evidence suggests that a shortened G1 phase is a typical feature of pluripotency, the causal relationship between them remains controversial. Specifically, whether the shortened G1 phase drives the maintenance of pluripotency or whether the pluripotent state actively shapes a brief G1 phase is debated. On one hand, some scholars argue that a shortened G1 phase itself constitutes the reason for maintaining pluripotency. Cells are more receptive to differentiation signals during G1, and their differentiation propensity changes as G1 progresses (Pauklin and Vallier, 2013). Therefore, shortening G1 can limit the time window during which cells respond to external differentiation cues, thereby passively “locking” cells into the pluripotent state. Studies have shown that artificially lengthening the G1 phase of mESCs through chemical or genetic means is sufficient to disrupt the pluripotency network and induce spontaneous differentiation, directly demonstrating that a short G1 phase is an intrinsic determinant of the naïve pluripotent state (Coronado et al., 2013). Conversely, enforced overexpression of *Cyclin E* or *CDK2* to actively shorten G1 can suppress differentiation programs and maintain the expression of pluripotency markers. In contrast, pharmacological inhibition of CDK2/Cyclin E activity to lengthen G1 promotes differentiation (Pauklin and Vallier, 2013).

Another important hypothesis holds that a short G1 is the consequence of core pluripotency factors regulating the cell cycle, rather than a driving force. Studies indicate that pluripotency transcription factors such as Sox2, Oct4, Klf4, and c-Myc actively promote G1 shortening by regulating the activity of Cyclin–CDK complexes and repressing *p21* expression, thereby promoting rapid proliferation, constraining the differentiation time window, and sustaining self-renewal (Fleifel and Cook, 2023). Moreover, the core pluripotency factor Nanog can directly bind and activate CDK6 and CDC25A, thereby promoting the G1/S transition (Boward et al., 2016). Consequently, G1 extension is a consequence of the differentiation program rather than a trigger for differentiation (Coronado et al., 2013).

Based on the aforementioned research, we propose that the short G1 phase and the pluripotent state are not in a simple one-way causal relationship, but are more likely to form a tightly coupled relationship (Dalton, 2015). This coupling manifests as a self-reinforcing, dynamically maintained feedback loop: the core pluripotency network actively configures the cell cycle toward a short G1 by repressing cell-cycle inhibitors and dismantling the DREAM complex; conversely, the short G1 indirectly limits exposure to extracellular differentiation signals while also enabling activated E2F transcription factors to co-occupy promoters with pluripotency factors, thereby creating an epigenetic and transcriptional environment that reinforces the pluripotent state. This bidirectional, interlocked coupling not only ensures self-renewal and rapid proliferation of PSCs but also substantially enhances their stability at differentiation thresholds. Consequently, the system operates as a robust whole: disrupting any link (whether by forcibly extending G1 or downregulating core pluripotency factors) would unravel the coupling and drive cells toward differentiation. Future studies that can dynamically track the interactions between pluripotency factors and cell-cycle regulatory elements in real time hold promise for more precisely delineating the temporal logic and hierarchical control of this coupling network in cell fate determination.

The universality of CDK1 as a core driver of the cell cycle is also debated. On one side, CDK1 is viewed as an indispensable “master switch” that can compensate for the loss of other CDKs. In nearly all somatic cells and most stem cells, complete inhibition of CDK1 leads to cell cycle arrest at G2/M. CRISPR-Cas9 screens also show that once CDK1 is activated, it is sufficient to drive cells into mitosis. Conversely, CDK1 inactivation directly causes G2/M arrest. This underscores the non-redundant nature of CDK1 for proliferation and survival (Santamaría et al., 2007). From an evolutionary perspective, CDK1 homologs in yeast act as the sole cell cycle CDKs controlling the entire cycle, underscoring its central role (Lee M. G. and Nurse, 1987). Another viewpoint does not deny the core role of CDK1 but suggests that its “absolute necessity” may be context-dependent and potentially possesses cell-cycle–independent functions. In certain cell types or states, other CDKs (such as CDK2) may assume more prominent roles. The necessity of CDK1 may vary with cellular metabolic state, DNA damage stress, or differentiation stage (Kozar et al., 2004). CDK1 loss leads to cell death, which may reflect not only cell-cycle arrest but also CDK1 involvement in transcriptional regulation, RNA splicing, DNA damage repair, and metabolism. Therefore, its “essentiality” likely reflects a combination of crisis due to cell-cycle arrest and disruption of key cellular processes. Additionally, technical limitations contribute to the controversy. Traditional gene knockout is long-term and complete; cells may activate compensatory or adaptive mechanisms during chronic CDK1 loss, confounding phenotypes. Acute, rapid knockout systems better reveal the most direct, primary functions.

In future studies, it will be valuable to precisely compare the speed and extent of cell-cycle arrest across different cell types after *CDK1* deletion. Constructing a CDK1 mutant that retains only non-Cyclin functions and assessing whether it can rescue all phenotypes caused by complete CDK1 loss would be of great significance for resolving the relationship between CDK1’s non-cyclin functions and stemness.

The functional redundancy and specificity of multi-subunit proteins have long been a focal point across disciplines. Cyclin D comprises Cyclin D1, Cyclin D2, and Cyclin D3. On one hand, Cyclin D1, D2, and D3 can all bind CDK4/6 and phosphorylate the same site on the Rb protein, thereby releasing its inhibition of E2F and promoting G1/S transition. This core biochemical function is highly conserved across the three subtypes. Studies have shown that placing the coding region of Cyclin D1 under the promoter control of Cyclin D2 can fully rescue the lymphoid developmental defects in *Cyclin D2*–deficient mice. This indicates that as long as Cyclin D1 protein is expressed, normal function can be performed, strongly supporting functional redundancy (Kalaszczynska et al., 2009). Conversely, other views argue that these Cyclin D subtypes have acquired unique functions during evolution. Expression profiles of Cyclin D subtypes differ: Cyclin D1 is highly expressed in most proliferative cells, Cyclin D2 in the hematopoietic system, and Cyclin D3 in early embryos and muscle (Sicinski et al., 1995). The phenotypes following individual deletions of *Cyclin D1*, *Cyclin D2*, or *Cyclin D3* are not identical, suggesting specific interactions with distinct transcription factors and kinases (Sicinska et al., 2003). Future studies on rescue experiments may provide a more comprehensive understanding of the functions of Cyclin D subtypes. For example, in mouse models with *Cyclin D2* and *Cyclin D3* double knockout, overexpression of *Cyclin D1* could be tested to determine whether it can fully rescue the defect phenotype. Proteomic analyses could identify the specific binding partners of each Cyclin D subtype. These subtype-specific interactors are likely the molecular basis for functional specificity.

The maintenance of pluripotency in ESCs and their unique cell cycle are not independent processes; they are interwoven through a robust bidirectional regulatory network. In this section, we synthesize extensive evidence showing that core cell cycle factors participate in stabilizing the pluripotency transcriptional network by directly phosphorylating and modifying proteins such as Sox2 and Oct4, affecting their activity, stability, or interactions. Conversely, core pluripotency factors Oct4, Sox2, and Nanog are not passive recipients; they actively shape rapid cell cycling. They function by transcriptionally repressing key CDK inhibitors and activating promoters of proliferation-promoting Cyclins. This mutual regulation forms a self-reinforcing feedback loop: the pluripotency network shapes a short G1 phase, and the short G1 phase, in turn, minimizes the time window for integrating differentiation signals and favors the establishment of an epigenetic landscape that supports self-renewal. This complex dialog ensures seamless coupling between rapid proliferation and the undifferentiated state.

### 2.2 The mechanisms of genomic stability maintenance in stem cells

Although the rapid proliferation of ESCs facilitates development, it also poses risks for genomic instability. To counteract this challenge, ESCs have evolved distinctive DNA damage response mechanisms, including Babam2’s role in stabilizing CDC25A and promoting the ubiquitination of p53, which helps to maintain CDK2 activity and preserve pluripotency factors (Biswas et al., 2018). The loss of *CDK1* results in increased DNA double-strand breaks (DSBs), mitotic abnormalities, and chromosomal aberrations, indicating genomic instability (Neganova et al., 2014; Wang X. Q. et al., 2017). *APE1* exerts its effects through a dual mechanism, specifically. On the one hand, it enhances the activity of the Gdnf/Gfrα1 axis, thereby activating the Src/Erk pathway to promote proliferation. On the other hand, it regulates the activity of Cyclin-CDK complexes to facilitate G1/S and G2/M phase progression, thereby preventing cell cycle arrest caused by frequent DNA damage (Liu L. et al., 2023; Malfatti et al., 2021). Simultaneously, ESC proliferation is accompanied by the accumulation of ROS(Wang K. et al., 2013). Dnm1L maintains ROS at physiological levels by regulating mitochondrial fission dynamics and the Keap1-Nrf2 antioxidant pathway, thus protecting genomic stability (Seo et al., 2023). Additionally, Cops5 regulates glycolytic metabolism by stabilizing mitochondrial transport protein Mtch2 and enhances DNA repair capacity, providing support for rapid cell cycle progression (Li P. et al., 2020).

ESCs with a shortened G1 phase are capable of rapid self-renewal, yet this may also result in insufficient assembly of the pre-replication complex (pre-RC) and inadequate nucleotide preparation. Such deficiencies can induce replication stress during accelerated DNA synthesis, leading to replication fork deceleration, stalling, or even collapse. These events ultimately cause DNA damage and genomic instability (Matson et al., 2017). To counteract this intrinsic challenge, ESCs have developed a sophisticated multilayered protective mechanism that is functionally linked to their pluripotent status. Low levels of basal replication stress, such as the formation of RPA-coated ssDNA, activate the ATR/CHK1 signaling pathway (Zhu X. F. et al., 2022). Once activated, ATR-CHK1 phosphorylates proteins including CLASPIN and FANCM to stabilize stalled replication forks and prevent their collapse. It also phosphorylates and stabilizes p53, which in turn induces p21 expression. As a broad-spectrum CDK inhibitor, p21 can initiate G1/S checkpoint arrest. Sustained activation of p53 not only enforces cell cycle arrest but also promotes the expression of differentiation-related genes while suppressing core pluripotency factors such as Nanog and Oct4. This facilitates the exit of ESCs from the pluripotent state (Lin T. et al., 2005). Under severe or irreparable stress conditions, p53 activation shifts toward inducing pro-apoptotic genes including *Puma* and *Bax*, thereby eliminating damaged cells. Furthermore, ESCs display heightened sensitivity to ATR-CHK1 inhibition, underscoring the critical role of this pathway in managing intrinsic replication stress. Inhibition of ATR or CHK1 results in markedly increased replication fork collapse and DNA damage, rapidly leading to apoptotic cell death. Additionally, studies have shown that various cellular stresses (including replication stress, oxidative stress, and osmotic stress) can induce a subfraction of ESCs to enter a 2-cell-like (2C-like) state. Cells in this state express 2C-stage–specific endogenous retroelements (such as *Mervl*) and display a more “naïve” epigenetic and transcriptional state, with a markedly slowed cell cycle (Oleksiewicz et al., 2017). It is hypothesized that this transition may be an evolutionarily conserved stress response pattern, wherein transcriptional programs are reprogrammed and cell cycle is slowed to cope with adverse conditions. When ESCs face severe stress, some cells survive by “rebooting” into a more primitive, protected state. A slowed cell cycle affords more time for DNA repair and metabolic adjustment.

In summary, ESCs employ a finely tuned, multilayered defense system to balance rapid proliferation with genomic integrity. The shortened G1 phase leads to inadequate replication preparation and increased replication conflicts, resulting in persistent basal replication stress. Consequently, ESCs depend critically on the ATR-CHK1 pathway for continuous monitoring and stabilization of replication forks. Depending on the severity of replication stress, cells activate distinct fate-determining mechanisms. Under mild stress, buffering networks—involving factors such as Babam2 and APE1—help maintain pluripotency by suppressing p53 and sustaining CDK activity. When stress escalates, strong activation of the p53–p21 axis induces cell cycle arrest, promotes differentiation, or initiates apoptosis to eliminate damaged cells and protect the stem cell pool. Under severe stress, certain cells may enter a 2C-like state through reprogramming, which slows the cell cycle and remodels the epigenome to facilitate survival. This multi-tiered response strategy collectively constitutes a core mechanism through which stem cells maintain genomic integrity and functional competence despite rapid proliferation.

Beyond their classical roles in driving cell cycle phase transitions, *Cyclins* and *CDKs* exhibit notable functional diversity and specificity in regulating stem cell fate. This section delves into these nonclassical functions of core regulatory factors, highlighting their direct involvement in epigenetic regulation, signal transduction integration, and lineage specification. We discuss how specific Cyclin-CDK complexes (e.g., Cyclin B1–CDK1) act as critical nodes linking G2/M to pluripotency maintenance. Additionally, this part examines functional redundancy and specificity among Cyclin subtypes and CDKs (e.g., the necessity of CDK1), emphasizing that their roles in the ESC context are highly context-dependent. The discussion also covers the key role of multi-protein complexes such as the DREAM complex, which acts as a master coordinator. It suppresses cell-cycle genes during quiescence and, during proliferation, ensures orderly, temporally specific expression of Cyclins and other factors, thereby correctly integrating developmental signals with core cell cycle operation to determine stem cell fate.

## 3 Signaling pathways connect the cell cycle and pluripotent genes in stem cells

The fate determination of stem cells is precisely regulated through multi-layered interactions among key signaling pathways and phase-specific cell cycle machinery (Guglielmi et al., 2021; Wulansari et al., 2021; Xu et al., 2023). Leukemia inhibitory factor (LIF) is a key factor in maintaining the pluripotency of mESCs. LIF binds to the cell surface receptor complex composed of LIF receptor (LIFR) and gp130, activating the Jak1 kinase, which then phosphorylates the transcription factor Stat3 (Darnell et al., 1994; Gearing et al., 1991; Stahl et al., 1994). Once phosphorylated, Stat3 dimerizes and translocates into the nucleus, where it directly binds to and activates the promoters of core pluripotency genes such as *Oct4*, *Sox2*, *Nanog*, and *Klf4* (Deng et al., 2021; Do et al., 2013; Li J. et al., 2024; Onishi and Zandstra, 2015; Stirparo et al., 2021). Recent studies have identified Gpr160 as an important regulator of the Jak1/Stat3 pathway; it enhances Stat3 phosphorylation and nuclear translocation, promoting the expression of CyclinD1-CDK4/6 and accelerating G1/S phase transition (Fan et al., 2024; Zhou C. H. et al., 2016). Notably, LIF not only activates the canonical Jak/Stat3 pathway but also bypasses other pathways to activate Erk1/2 and PI3K/Akt signaling networks, forming a complex regulatory system (Boulton et al., 1994; Oh et al., 1998).

MAGEA2 (Melanoma-Associated Antigen A2) contributes to sustaining stem cell pluripotency and promoting proliferation, in conjunction with other core pluripotency factors such as Oct4, Sox2, and Nanog (Barker and Salehi, 2002; Lifantseva et al., 2011; Yang B. et al., 2007). Studies have found that knockdown of *MAGEA2* leads to increased phosphorylation of Erk1/2, and decreased expression of cell cycle-related genes such as *CDK1*, *CDK2*, *CyclinA1*, *CyclinD1*, and *CDC25a*. These molecular changes result in cell cycle arrest, accompanied by a reduction in the expression of pluripotency markers in mESCs. These findings suggest that the MAGEA2/Erk1/2 signaling pathway plays a crucial role in both maintaining the expression of core pluripotency genes and cell cycle genes, as well as inhibiting embryonic lineage differentiation (Park et al., 2020).

Under energy stress conditions, the AMPK signaling pathway exhibits a bidirectional role in the regulation of ESC pluripotency, depending on the cellular state. In pluripotent cells, AMPK inhibits *Nanog* transcription and promotes its proteasomal degradation *via* the p53/p21 pathway, driving differentiation. Conversely, in the absence of LIF or under low pluripotency conditions, AMPK stabilizes β-catenin through PI3K/Akt-mediated inhibition of GSK3β, thereby activating the Wnt pathway. The activated β-catenin forms a transcriptional complex with Oct4 and cooperatively upregulates naive pluripotency factors such as Klf4 and Esrrb, significantly increasing *Nanog* expression. Simultaneously, AMPK induces G1 phase arrest and G2/M phase suppression; through p21-dependent cell cycle reprogramming, it slows proliferation, providing a temporal window for the re-establishment of pluripotency transcription factors (Alba et al., 2020; Chae et al., 2012; Liu Y. J. and Yamashita, 2019). This metabolic-cycle-pluripotency tri-regulatory network dynamically coordinates ESC fate decisions under energy stress, enabling adaptation to the environment.

The Hippo/YAP-TAZ pathway modulates the balance between proliferation and differentiation in a cell cycle phase-dependent manner. In trophoblast stem cells, nuclear localized YAP interacts *via* its WW2 domain with the stemness factor Cdx2*,* represses the expression of the G1-phase regulator Cyclin D1, and reduces CDK4/6 activity, thereby restraining the G1 progression rate. This “YAP–CDX2–Cyclin D1” axis specifically maps to G1-phase regulation (Basak and Ain, 2022). In the G2/M phase or endoreplication stage, downregulation of the core kinase LATS1 attenuates the formation of the LATS1–LIMK2 complex, relieving inhibition of LIMK2 and leading to elevated pLIMK2Thr505 and subsequent pCOFILINSer3 levels. This stabilizes F-actin and promotes endoreplication (polyploidization) in trophoblast giant cells, corresponding to the G2/M-to-endoreplication transition (Basak and Ain, 2022).

The Notch pathway antagonizes Hippo/YAP-TAZ and is linked to G1 exit. In epidermal stem cells, YAP/TAZ transcriptionally activate Delta-like ligands (DLL1, DLL3), which inhibit Notch signaling in a cis-inhibitory manner to maintain stemness. Upon YAP/TAZ inactivation—triggered by soft substrates or high cell density—Notch signaling is activated, inducing G1 exit and initiating differentiation. This switch depends on the transcriptional control of Notch ligands by YAP/TAZ and is explicitly associated with the transition between stemness maintenance and differentiation in G1 (Totaro et al., 2017).

Phase-specific CDK activities act as downstream effectors of these pathways: G1 CDK4/6 activity is directly suppressed by YAP, whereas dysregulated CDK activity during endoreplication is modulated indirectly *via* the LATS1–LIMK2 axis. Notch activation likely promotes cell cycle exit and differentiation by downregulating G1 Cyclin–CDK complexes (Basak and Ain, 2022; Meyer et al., 2023; Totaro et al., 2017). Furthermore, oscillatory YAP activation optimizes G1 CDK-mediated proliferation, while sustained low YAP activity synergistically promotes differentiation through Notch activation and CDK downregulation, reinforcing the phase-specific mapping of pathway activities (Meyer et al., 2023).

The TGF-β/Activin/Nodal–Smad pathway cooperates with cell cycle regulators to control pluripotency maintenance and lineage specification. Early in G1, when Cyclin D–CDK4/6 activity is low, Smad2/3 translocate into the nucleus to bind endodermal gene promoters and initiate differentiation programs. Concurrently, they upregulate the DNA methyltransferase *Dnmt3b* to establish methylation patterns, facilitating the binding to epiblast markers such as Fgf5 and Dnmt3a. This Smad4-independent process is critical for the transition from naive to primed pluripotency (Zhao et al., 2024). By late G1, elevated Cyclin D–CDK4/6 activity phosphorylates the linker regions of Smad2/3, inhibiting their nuclear translocation and thereby diverting cells toward neuroectodermal differentiation instead of Activin/Nodal-induced endodermal commitment (Pauklin and Vallier, 2013; Yang J. and Jiang, 2020). Additionally, Nodal/Activin signaling regulates the dosage of pSmad2, which binds the *Oct4* promoter in a concentration-dependent manner to sustain the pluripotency network (Lee K. L. et al., 2011). Smad4 functions primarily during primed-to-mesendodermal differentiation, forming complexes with Smad2/3 to activate lineage-specific genes such as *Wnt3* and *Eomes* (Zhao et al., 2024). The PI3K/Akt pathway further fine-tunes this balance *via* mTORC2-mediated degradation of Smad2/3, antagonizing their differentiation-inducing effects (Yang J. and Jiang, 2020).

The Wnt/β-catenin pathway contributes to stem cell regulation by maintaining epigenetic stability. Its decline accelerates the cell cycle, impairs proper pluripotency exit, and compromises differentiation potential. Mechanistically, β-catenin cooperates with the KAP1/DNMT1 complex to maintain DNA methylation and heterochromatic states at imprinting control regions (ICRs), suppressing retrotransposon activity and ensuring genomic stability and cellular homeostasis (Theka et al., 2019).

The Hedgehog (HH) pathway orchestrates NSC dynamics through its downstream GLI transcription factors (particularly GLI1 and GLI2). It shortens the cell cycle of activated NSCs (aNSCs) by accelerating both G1 and S/G2/M phases, thereby enhancing proliferation and self-renewal capacity, as reflected by increased neurosphere formation (Daynac et al., 2016). Moreover, GLI2 directly binds to *Sox2* enhancers to drive its expression in NSCs. *Sox2* in turn activates *Hes5*, contributing to the maintenance of an undifferentiated state (Takanaga et al., 2009). However, prolonged HH activation induces accumulation of quiescent NSCs (qNSCs) but ultimately exhausts the aNSC pool, leading to loss of pluripotency, premature cell cycle exit, and aberrant differentiation, underscoring its dual role in NSC homeostasis (Agathocleous et al., 2007; Daynac et al., 2016).

Signal transduction pathways are the key bridge connecting extracellular signals to intracellular responses. They seamlessly integrate environmental cues with core cell cycle control and pluripotency. This section explains how major signaling pathways—including LIF/Jak/Stat3, PI3K/Akt, TGF-β/Smad, Wnt/β-catenin, and Hippo/YAP—orchestrate stem cell fate by directly regulating the activity of Cyclin-CDK complexes, CKIs, and pluripotency transcription factors. These pathways are not independent. They are interwoven and exhibit temporal specificity. The Hippo/YAP pathway can produce opposite effects depending on the relative expression of *Cyclins* and *CDKs* at different cell cycle phases, while TGF-β/Smad signaling is gated by G1-phase CDK activity. In addition, AMPK signaling can bidirectionally influence pluripotency by affecting the cell cycle and key transcriptional networks such as Wnt/β-catenin. This complex interplay ensures that decisions of self-renewal and differentiation are precisely calibrated according to cellular metabolic state and the external microenvironment.

## 4 Linking of cell cycle and epigenetic modification in ESCs

Epigenetic regulation also plays a crucial role in maintaining the cell cycle characteristics of ESCs(Roy et al., 2022). The enrichment patterns of histone modifications, such as H3K27ac and H3K4me3, at the promoters of cell cycle genes vary significantly between ESCs and somatic cells. In particular, the promoters of pluripotency-related genes maintain an “open” chromatin conformation in ESCs, enabling rapid responsiveness to environmental signals and facilitating adjustments to the cell cycle.

Studies have shown that deletion of Jmjd2 family proteins, which are histone demethylases, either individually or collectively, leads to impaired ESC self-renewal and early embryonic lethality. Their primary role involves removing H3K9 methylation to ensure pluripotency (Pedersen et al., 2016). The Polycomb Group (PcG), comprising PRC1 and PRC2, is a highly conserved epigenetic repressive complex throughout evolution. PRC1 catalyzes monoubiquitination of histone H2A at lysine 119 (H2AK119ub1), while PRC2 mediates trimethylation of histone H3 at lysine 27 (H3K27me3), jointly establishing a repressive chromatin state that silences differentiation-related genes (Qin et al., 2021; Zhu Y. R. et al., 2022). Research indicates that PRC1 and PRC2 also promote the maintenance of the short G1 phase characteristic of ESCs by repressing the expression of G1 extension-related genes, such as *p21*, and by enhancing the activity of the CyclinE/CDK2 complex (Huang et al., 2021; Zhu Y. R. et al., 2022). DNA demethylase Tet1 collaborates with PRC2 to remove DNA methylation marks and enhance H3K27me3 modification, forming a multilayered epigenetic regulatory network. This synergistic effect is crucial for maintaining the activity of pluripotency genes and coordinating the balance between proliferation and differentiation (Chrysanthou et al., 2022a; Chrysanthou et al., 2022b; Ficz et al., 2011).

The unique cell cycle of ESCs is both a cause and a consequence of their distinctive epigenetic features. We discusses the deep bidirectional relationship between the epigenome and the cell cycle in this section. Key epigenetic modifiers, such as Polycomb repressive complexes (PRC1/2) and histone demethylases, are essential for maintaining a shortened G1 phase by repressing the expression of cell cycle inhibitors and pro-differentiation genes. Conversely, core cell cycle regulatory proteins participate in maintaining these epigenetic characteristics. CDKs can directly phosphorylate epigenetic enzymes, altering their activity and affecting histone modification patterns that are critical for fate decisions. This coordinated regulatory loop ensures ESCs proliferate rapidly while maintaining an epigenetically reinforced pluripotent state and suppressing premature differentiation, indicating that epigenetic regulation is a dynamic process tightly synchronized with the cell cycle.

## 5 Role of metabolic mode in stem cells

ESCs exhibit distinctive metabolic characteristics that provide energy and biosynthetic precursors necessary for rapid proliferation. ESCs primarily depend on glycolysis for energy production. This metabolic pattern not only meets the energetic demands of rapid cell division but also regulates histone acetylation and gene expression by modulating levels of metabolic intermediates such as acetyl-CoA. Recent studies have identified the mTOR signaling pathway as a key hub integrating nutrient signals with cell cycle regulation. Reducing the activity of the mTOR pathway can induce hPSCs and blastocysts to enter a quiescent state, in which cell proliferation, developmental processes, and attachment to the uterine epithelium are all limited (Iyer et al., 2024). Sustained activation of *mTORC1* promotes protein synthesis and cell growth, providing the material foundation for frequent cell division (Joshi et al., 2024). Meanwhile, *mTORC2* influences mitosis by regulating cytoskeletal reorganization (Palma et al., 2023). This metabolism-cycle coupling mechanism ensures that ESCs can rapidly expand during early embryonic development.

Even under high oxygen conditions, ESCs mainly rely on glycolysis rather than (OXPHOS) for ATP production. Studies have shown that the long non-coding RNA (lncRNA) Lx8-SINE B2 enhances glycolytic flux by binding to the glycolytic enzyme Enolase 1 (ENO1), stabilizing and activating ENO1, thereby significantly increasing glucose utilization. When glycolysis is inhibited, ATP supply becomes insufficient, leading to decreased activity of Cyclin-CDK complexes, delayed G1/S phase transition, and ultimately the loss of pluripotency (Chen F. Q. et al., 2022b; Chen F. Q. et al., 2021a).

Stem cell metabolism constitutes a sophisticated regulatory network that governs energy supply, epigenetic modifications, and the elimination of toxic metabolites to maintain pluripotency and cell cycle homeostasis. This network exhibits state-specific and species-dependent characteristics. Ground-state PSCs, such as mESCs cultured in 2i/LIF conditions (mESCs-2iL) and human induced pluripotent stem cells (iPSCs), predominantly rely on a high glycolytic flux. Even in the presence of homologous or heterologous mitochondrial DNA (mtDNA) mutations, human iPSCs sustain elevated expression of key glycolytic genes, including the glucose transporter *Glut3* and hexokinase HK3. Moreover, they suppress pyruvate dehydrogenase (PDH) activity *via* upregulation of pyruvate dehydrogenase kinase 1 (PDK1), thereby preventing the entry of pyruvate into the tricarboxylic acid (TCA) cycle and promoting the glycolytic pathway. This metabolic preference not only facilitates rapid ATP generation to meet the proliferative demands of the short G1 phase but also minimizes ROS accumulation from OXPHOS, thereby avoiding DNA damage and senescence pathway activation. Consequently, it helps stabilize the expression of core pluripotency factors (Prigione et al., 2014). The PTEN-induced kinase 1 (PINK1)-mediated mitophagy is a critical mechanism enabling this glycolytic switch. Loss of PINK1 leads to accumulation of damaged mitochondria, increased ROS levels, and activation of the p53-dependent cell cycle checkpoint, thereby causing an 80% reduction in reprogramming efficiency and a 3–4 days delay in the emergence of SSEA-1-positive colonies in mESCs. In contrast, functional mitophagy eliminates mature tubular mitochondria and promotes the formation of immature spherical mitochondria, providing the structural basis for a glycolytic metabolic profile (Vazquez-Martin et al., 2016).

Acetyl-CoA serves as a central metabolic effector of pluripotency. Its cellular level and subcellular distribution profoundly influence stem cell fate by modulating histone acetylation and pluripotency gene expression. Both mouse and human PSCs require high acetyl-CoA levels to support active histone marks such as H3K9ac and H3K27ac, as well as an open chromatin configuration. mESCs primarily generate acetyl-CoA through threonine metabolism, while hPSCs rely on glucose-derived pyruvate, which is converted to citrate and transported to the cytoplasm, where ATP-citrate lyase (ACLY) catalyzes its conversion to acetyl-CoA. Exogenous acetate supplementation increases acetyl-CoA levels and delays differentiation of PSCs(Chakrabarty and Chandel, 2021). NAD+ promotes the deacetylation and activation of acetyl-CoA synthetase 1 (AceCS1) *via* Sirt1, facilitating acetate conversion to acetyl-CoA, thereby maintaining acetyl-CoA and H3K27ac levels in mESCs and supporting pluripotency (Wu et al., 2022). Additionally, nuclear-localized TCA cycle enzymes, such as Pdha1, can directly enhance the nuclear acetyl-CoA pool, augment histone H3 acetylation, activate pluripotency genes including *Oct4* and *Nanog*, and promote somatic reprogramming as well as the naïve-to-primed transition (Li et al., 2022a). In NSCs, TIGAR enhances OXPHOS, elevates acetyl-CoA levels, increases H3K9ac modification, and activates differentiation-related genes such as *Ngn1* and *Neurod1*, thereby regulating neurogenic progression (Zhou W. et al., 2019).

One-carbon metabolism represents a core pathway linking metabolic flux to epigenetic regulation of pluripotency, with notable species-specific variations. mESCs depend on threonine (Thr) metabolism to obtain one-carbon units: Thr is catabolized by threonine dehydrogenase (TDH) to generate glycine and acetyl-CoA. Glycine is further metabolized to formate, which enters the S-adenosylmethionine (SAM) cycle to provide methyl donors for histone H3K4me2 and H3K4me3 modifications. These marks are enriched at the promoters of pluripotency genes such as *Esrrb* and *Klf4*, maintaining an open chromatin state. Threonine deprivation leads to a significant reduction in H3K4me2/3 levels, causing proliferation arrest and initiation of differentiation in mESCs, without affecting DNA methylation or other histone modifications such as H3K9me3 (Shyh-Chang et al., 2013; Van Winkle and Ryznar, 2019). In contrast, hESCs, which lack functional TDH, depend on methionine (Met) metabolism. Met cycles through the Met-SAM pathway to generate SAM, which serves as the methyl donor for H3K4me3 and DNA methylation. Methionine deficiency results in near-complete loss of H3K4me3, proliferation arrest, and apoptosis in hESCs, phenotypes that can be partially rescued by exogenous SAM supplementation (Shiraki et al., 2014; Van Winkle and Ryznar, 2019). Furthermore, the glycine cleavage system (GCS) is highly active in PSCs. Its rate-limiting enzyme, glycine decarboxylase (Gldc), is co-regulated by Sox2 and Lin28A. GCS generates one-carbon units *via* glycine cleavage to support SAM synthesis and maintain H3K4me3 levels, while also clearing methylglyoxal (MG), a toxic byproduct of aberrant glycine metabolism. This prevents accumulation of advanced glycation end products (AGEs) and activation of senescence markers (P15, P16, P21), thereby avoiding irreversible G1 arrest and ensuring normal cell cycle progression (Tian et al., 2019).

Alpha-ketoglutarate (α-KG), a key intermediate of the TCA cycle, is an important regulator of pluripotency. In mESCs, α-KG is primarily generated by mitochondrial isocitrate dehydrogenase 2 (IDH2). Exogenous supplementation with cell-permeable dimethyl-α-ketoglutarate (dm-αKG) can substitute for 2i inhibitors (GSK3 and MEK inhibitors) in maintaining ground-state pluripotency. dm-αKG promotes DNA demethylation by activating TET dioxygenases and reduces H3K9me2 levels *via* Kdm3a/b histone demethylases, thereby sustaining the proportion of Rex1-GFP-positive cells and dome-shaped colony morphology. During differentiation, dm-αKG extends the developmental competence window of epiblast-like cells (EpiLCs) for differentiation into primordial germ cell-like cells (PGCLCs), enabling EpiLCs cultured for 72 h to differentiate into PGCLCs with efficiency comparable to that of 48-h controls, while maintaining H3K27me3 levels to ensure precise activation of key PGC genes such as *Prdm1* and *Prdm14* (Carey et al., 2015; Tischler et al., 2019). The transcription factor Spic further reinforces the regulation of ground-state pluripotency by one-carbon metabolism. In 2iL culture, upon inhibition of MEK/Erk signaling, Spic is transcriptionally activated and stabilizes Nanog binding at chromatin regions of betaine-dependent one-carbon metabolism genes, enhancing betaine-to-methionine conversion and maintaining a low SAM/SAH ratio. This process upregulates activating histone marks such as H3R17me2a while suppressing H3K4me3 (to avoid differentiation gene activation), thereby delaying exit from ground-state pluripotency. Concurrently, it provides precursors for dTMP synthesis and other DNA biosynthesis requirements, enhancing cell cycle adaptability under metabolic stress (Azad et al., 2023).

The maintenance of pluripotency is closely associated with mitochondrial remodeling and metabolic reprogramming. Despite the potential presence of homologous or heteroplasmic mtDNA mutations, hiPSCs maintain a highly glycolytic phenotype similar to hESCs by upregulating glycolytic enzymes, increasing glucose-6-phosphate (G6P) levels, and enhancing PDK1 expression (Prigione et al., 2014). Naïve-state ESCs and iPSCs possess immature, spherical mitochondria with underdeveloped cristae and rely predominantly on glycolysis. Upon differentiation into NSCs, mitochondria become elongated with well-defined cristae, and cells shift toward OXPHOS(Choi et al., 2015). Additionally, PINK1-mediated mitophagy promotes mitochondrial rejuvenation and is essential for efficient reprogramming. Its loss significantly impairs reprogramming efficiency, diminishes glycolytic capacity, and reduces α-KG production, thereby compromising pluripotency establishment (Vazquez-Martin et al., 2016).

ESCs rely heavily on glycolytic metabolism, which is not merely a passive adaptation to rapid proliferation but an active regulator of pluripotency and cell cycle progression. This section clarifies how metabolism, the cell cycle, and pluripotency are precisely coupled. A high glycolytic flux provides ample biosynthetic precursors and ATP while maintaining low ROS, supporting rapid biomass accumulation and minimizing DNA damage. Importantly, key metabolic pathways and intermediates directly influence epigenetic modifiers and signaling. Metabolites such as acetyl-CoA, α-ketoglutarate (α-KG), and S-adenosylmethionine (SAM) are essential cofactors for histone acetylation and methylation, directly linking cellular metabolism to the epigenetic regulation of pluripotency genes. Moreover, species-specific metabolic dependencies (e.g., threonine metabolism in mESCs and methionine cycling in hESCs) underscore the evolutionary adaptability of this coupling. Sensors such as AMPK and mTOR integrate energy status with cell cycle decisions, regulating CDK activity and the stability of pluripotency factors. Thus, metabolism acts as a central hub, coordinating energy production, biosynthetic demands, and epigenetic signaling to sustain self-renewal and rapid proliferation.

## 6 Cell cycle control and somatic cell reprogramming

The breakthroughs in somatic cell reprogramming have effectively enabled the regulation of cell fate, with significant implications for clinical applications, drug screening, *in vitro* modeling of human diseases, and understanding the early stages of pre-implantation embryonic development. Somatic cell reprogramming refers to the process of reversing mature somatic cells to a pluripotent, embryo-like state or iPSCs by introducing specific transcription factors. This process involves not only reorganization of transcriptional networks but also profound reconfiguration of cell cycle proteins and pluripotent factors. Gurdon and colleagues were the first to discover the method of reprogramming through somatic cell nuclear transfer, which resets the somatic cell nucleus during fertilization by oocyte factors (Campbell et al., 1996; Gurdon, 1962; Washburn et al., 2022).

Yamanaka and colleagues identified the so-called “Yamanaka factors”—Oct3/4, Sox2, c-Myc, and Klf4—from a pool of 24 candidates. The successful reprogramming of fibroblasts into iPSCs with these four factors culminated in the 2012 Nobel Prize in Physiology or Medicine. This landmark discovery opened the door to reprogramming somatic cells into PSCs using defined factors (Takahashi and Yamanaka, 2006). One of the main objectives of cell fate manipulation in reprogramming is to expand the pool of scarce cell sources, and as reprogramming techniques and methods become increasingly refined, these functional cells are expected to be used in the treatment of various major diseases. Moreover, somatic cell reprogramming provides an unlimited source of PSCs for cell replacement therapies, circumventing the ethical issues associated with embryo destruction, and enables the construction of disease models using patient-specific iPSCs to investigate pathogenic mechanisms (Wattanapanitch, 2019).

**FIGURE 2:**
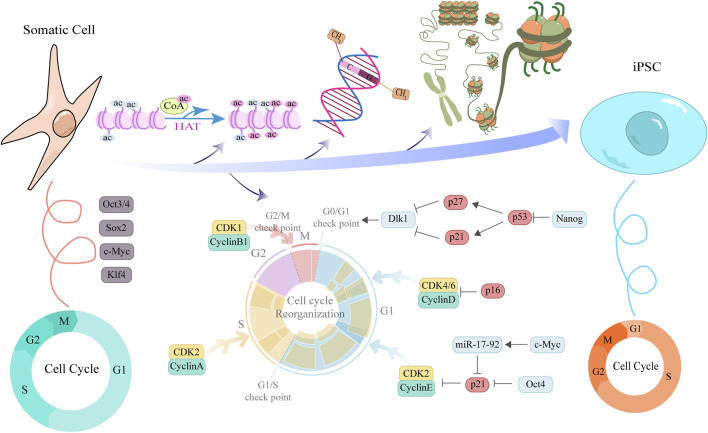
Schematic illustration of cell cycle remodeling and associated factors function during somatic cell reprogramming to iPSCs. This diagram elucidates the molecular mechanisms by which cyclins and pluripotency factors interact to promote the reprogramming of somatic cells into iPSCs. The upper section depicts key epigenetic events underlying the transition from a tightly compacted to an open chromatin structure during reprogramming, including the regulation of histone acetylation, DNA methylation, and chromatin remodeling. The central portion illustrates the ongoing cell cycle remodeling, highlighting the dynamic expression of cyclins across distinct cell cycle phases. Cyclin expression facilitates cell cycle progression and contributes to pluripotency maintenance, whereas CKIs act at cell cycle checkpoints to modulate reprogramming efficiency. Concentric ring diagrams flanking the central illustration provide a comparative analysis of cell cycle structural features between somatic cells and PSCs, underscoring the pivotal role of cell cycle remodeling in the somatic cell reprogramming process.

Increasing evidence indicates that somatic cell reprogramming is not simply the overexpression of four transcription factors (OSKM), but a dramatic identity conversion process impeded by multiple barriers (Figure 2). The cell cycle regulatory network is one of the most critical gatekeeping mechanisms. It not only limits the speed of reprogramming but also acts as a quality controller, eliminating cells that fail to meet criteria. Successful reprogramming is accompanied by profound remodeling of the cell cycle, enabling escape from these intrinsic inhibitory barriers. In normal somatic cells, the cell cycle is composed of tightly regulated phases, including G1, S, G2, and M phases, with Cyclins and CDKs collaboratively controlling the process. During their maintenance of division and metabolic homeostasis, somatic cells typically exhibit a prolonged G1 phase and a relatively slow cell cycle to ensure accurate DNA replication and cellular stability. Studies have shown that cells in the quiescent G0 phase usually display low levels of Cyclin D and E expression, with reduced CDK activity, making entry into the S phase difficult (Liu et al., 2019).

In contrast, during reprogramming—especially in the later stages—dedifferentiated cells acquire the distinctive cell cycle features of PSCs, including a shortened G1 phase and an accelerated proliferation rate. These features support the restoration of a high proliferative state, which facilitates—but is not solely sufficient for—the establishment of pluripotency (Ruiz et al., 2011). The restructuring of the cell cycle is particularly pronounced in somatic cell reprogramming (Cacchiarelli et al., 2015). A team has proposed a reprogramming strategy in which the inhibition of cell cycle inhibitors (such as p27 or p18) in somatic cells significantly enhances reprogramming efficiency (Zhan et al., 2019). This is primarily because these inhibitors play a key role in maintaining cell quiescence and the early G1 phase, thereby hindering entry into the S phase. Blocking these inhibitory signals can promote cells to advance, shortening the G1 phase, accelerating cell proliferation, and rapidly accumulating the expression of pluripotency transcription factors. Studies have shown that, among the four reprogramming factors proposed by Yamanaka, Myc is most closely associated with cell cycle regulation (Daksis et al., 1994; Washburn et al., 2022). Consistently, overexpression of *CyclinB1-CDK1* (Wang X. Q. et al., 2017; Yang G. Q. et al., 2023), *CyclinD-CDK4/6*, or *CyclinE/A-CDK2*, as well as downregulation of *pRb*, can increase reprogramming efficiency by promoting rapid cell proliferation (Ruiz et al., 2011; Zhan et al., 2019). Conversely, ectopic expression of cell cycle inhibitors such as *p15*, *p16*, or *p21* (Ruiz et al., 2011), or use of small-molecule inhibitors targeting CDK2 or CDK4 (Zhan et al., 2019), impedes cell cycle progression and reduces reprogramming efficiency. CDK2-mediated phosphorylation of Sox2 at Ser-39/Ser-253 is essential for reprogramming mouse embryo fibroblasts (MEFs) to a pluripotent state but is not required for maintaining mESCs(Ouyang et al., 2015). The p53 pathway is the central guardian of genomic integrity in somatic cells and is likewise activated to cope with the drastic cellular state remodeling and consequent replication stress during reprogramming. Ectopic expression of OSKM directly induces DNA damage and replication stress, thereby activating *p53*. *p53* upregulates *p21* transcriptionally, and p21, as a broad-spectrum CKI, induces G1 arrest. This provides the cells with a window to repair DNA damage or to initiate apoptosis, thereby effectively preventing the emergence of potentially mutated cells, but at the cost of substantially reduced reprogramming efficiency (Marión R. M. et al., 2009a). p16 is another important senescence-associated CDK inhibitor that maintains the tumor-suppressive function of the Rb protein by specifically inhibiting CDK4/6, thus triggering G1 arrest. In senescent or aged donor somatic cells, *p16* expression is higher, constituting an age-related barrier to reprogramming efficiency (Li H. et al., 2009). The intact functions of p53 and p16 are crucial to ensuring the genomic quality of iPSCs. Transient inhibition of the p53–p21 axis or p16 can markedly improve reprogramming efficiency by allowing more cells to pass the G1/S checkpoint and enter cell-cycle states that are more conducive to reprogramming. Complete ablation of their function, while it can dramatically increase efficiency, leads to iPSC clones with genomic instability and a significantly heightened risk of tumorigenicity. Therefore, during reprogramming, the ideal strategy is to transiently dampen rather than completely ablate the functions of p53–p21 and p16. Most somatic cells have short telomeres and low telomerase (TERT) activity.

The aggressive proliferation induced by OSKM accelerates telomere shortening and telomere dysfunction, which is recognized by the cell as DNA damage and similarly activates the p53–p21 pathway, eliciting senescence or apoptosis (Marion et al., 2009b). The Shelterin complex serves as the protective cap of telomeres. During reprogramming, alterations occur in the expression and function of Shelterin components. As a result, exposed telomeres are recognized as double-strand breaks. This recognition activates the ATM/ATR–mediated DNA damage response pathway. In connection with reprogramming, co-expression of *TERT* can mitigate telomere erosion, lessen the DNA damage response, and thereby improve reprogramming efficiency, particularly in late-passage somatic cells. Studies show that OSKM itself upregulates *TERT* and certain Shelterin proteins, and successful reprogramming is accompanied by telomere elongation and restoration of function, marking a key step toward achieving true pluripotency.

DNA replication occurs in the S phase of the cell cycle. However, replication is not uniform but follows a strict spatiotemporal order. The replication timing (RT) is highly correlated with the three-dimensional (3D) chromatin structure and transcriptional activity. Somatic cells exhibit RT patterns that are unique to their cell type. PSCs exhibit another distinct, tightly coordinated RT pattern, characterized by more early-replication domains and clearer temporal switches (Marion et al., 2009b). In the reprogramming process, remodeling of RT is a late-stage event, yet it is crucial (Matson et al., 2017). Resetting the RT pattern of somatic cells to a pluripotent-state RT pattern embodies and preconditions comprehensive epigenomic reorganization. Incomplete RT remodeling leads to fork progression at the wrong time and place, exacerbating transcription–replication conflicts (TRCs), thereby triggering replication stress, DNA double-strand breaks, and genomic instability. This renders the S phase a potential bottleneck for reprogramming failure. Efficient reprogramming requires the coordination of RT remodeling with epigenetic modifications and changes in 3D genome architecture. CDK activity directly influences chromatin structure and the selection of replication origins by phosphorylating histones, chromatin regulators, and replication initiation proteins, thereby indirectly regulating the remodeling of RT. Moreover, CDK kinase activity participates in a positive feedback loop with pluripotency factors, directly catalyzing the reprogramming process. CDK1/2-mediated phosphorylation of Oct4 enhances its transcriptional activity and protein stability. CDK1/2-mediated phosphorylation of Sox2 affects its DNA-binding capacity and transcriptional activity. OSKM also exerts feedback regulation on CDKs: the OSKM factors upregulate genes that promote cell cycle progression while suppressing CDK inhibitors such as p16 and p21, thereby actively creating a proliferation-promoting cell-cycle environment. The use of small-molecule CDK inhibitors or CKIs can modulate the cell cycle to indirectly influence reprogramming efficiency. This synergistic interaction forms a positive feedback loop: OSKM promotes cell cycle progression, enabling more cells to enter a permissive state, and CDK-mediated phosphorylation enhances OSKM activity, leading to more efficient reprogramming.

Alterations in the expression of pluripotency factors also play a central role in cell cycle restructuring during reprogramming. During the initial phase of reprogramming, the expression levels of exogenous transcription factors such as Oct4, Sox2, Klf4, and Nanog gradually increase. Studies indicate that core pluripotency factors not only activate pluripotency-related genes but also influence cell cycle status by regulating the expression of cell cycle proteins. For example, Oct4 has been shown to suppress *p21* expression, thereby promoting entry into the S phase and accelerating cell division to improve proliferative efficiency (Lu Y. et al., 2019). c-Myc-driven upregulation of miR-17-92 enhances reprogramming efficiency by targeting *p21* for inhibition (Ishida et al., 2023). Knockdown of *Nanog* significantly inhibits cell proliferation by upregulating *p27* and *p21 via* the p53 pathway, leading to G0/G1 phase cell cycle arrest and regulation of *Dlk1*. Furthermore, Nanog suppression reduces *Dnmt1* expression, which also be involved in cell cycle regulation (Pitrone et al., 2019). Additional studies have demonstrated that upon radiation-induced DNA damage, Klf4 can inhibit the expression of *CyclinD1* and *CyclinB1*, thereby blocking the transition of the cell cycle at both the G1/S and G2/M checkpoints. This highlights the critical role of Klf4 in maintaining the integrity of the G2/M checkpoint following DNA damage (Yoon and Yang, 2004). These pluripotency factors modulate cell cycle protein expression and are reciprocally regulated by cell cycle states, forming a bidirectional control system that ensures sufficient proliferation capacity while activating pluripotency genes to complete reprogramming.

Furthermore, the restructuring of the cell cycle during reprogramming involves epigenetic regulation. Activation of epigenetic modifications—such as histone deacetylation, DNA demethylation, and the establishment of an open chromatin state—contributes to the activation and expression of pluripotency genes (Guo et al., 2025). In this process, the cooperation between cell cycle proteins and pluripotency factors facilitates a stably reprogrammed state by modulating the cell cycle, thereby maintaining chromatin in a transcriptionally permissive configuration conducive to the establishment of pluripotency.

It is important to note that while cell cycle reprogramming enhances reprogramming efficiency, it also poses potential risks of genomic instability. Rapidly proliferating cell states are prone to chromosomal breaks, aneuploidy, and mutation accumulation, thereby increasing the risk of genomic damage. Consequently, balancing rapid cell proliferation with the stability of genomic integrity has become a key focus of current research. During reprogramming, by regulating the expression of cell cycle proteins and simultaneously enhancing pathways involved in DNA repair and chromosome stability, reprogramming efficiency can be improved while reducing the risk of genomic instability.

In summary, cell cycle reprogramming during somatic cell reprogramming is jointly driven by reciprocal regulation between cell cycle proteins and pluripotency factors. The upregulation of cell cycle proteins such as CyclinD, CyclinE, and CyclinA, combined with the activation of pluripotency factors, promotes a rapid entry into the S phase, facilitating the transition of cells from a quiescent to a highly proliferative state. This process relies not only on the regulation of cell cycle protein expression but also involves the activation of pluripotency gene networks, which form positive feedback loops to reinforce the pluripotent state. Furthermore, epigenetic modifications further promote chromatin accessibility and the expression of pluripotency genes, significantly enhancing reprogramming efficiency. However, the rapid proliferation associated with this process also increases the risk of genomic instability. Researchers are continuously exploring strategies to maintain genomic integrity while achieving high reprogramming efficiency, aiming to enable the safer and more effective transformation of somatic cells into PSCs. These studies not only reveal the complex relationship between cell cycle proteins and pluripotency factors but also provide a theoretical foundation and practical guidance for optimizing reprogramming technologies and developing safer cell-based therapies. The p53 pathway is a major barrier to cellular reprogramming, and researchers have explored transient inhibition of the p53 pathway with small molecules during reprogramming and withdrawal of these compounds in the late stage to improve reprogramming efficiency without overly compromising genomic safeguarding functions (Marión et al., 2009a). In addition, Vitamin C, a commonly used antioxidant, can serve as a cofactor for epigenetic modifiers (e.g., promoting histone demethylation) to remodel the epigenome, alleviating oxidative stress and associated DNA damage, thereby generating higher-quality iPSCs more efficiently (Esteban et al., 2010). Non-integrating gene delivery systems (such as episomal plasmids or mRNA transfection) are developed to avoid insertional mutations and ensure genomic stability (Yu J. et al., 2009). These strategies aim to balance reprogramming efficiency with safety, producing high-quality iPSCs for clinical applications.

Somatic cell reprogramming is accompanied by remodeling of the somatic cell cycle, endowing cells with cycle characteristics similar to those of PSCs. Synthesizing prior research, we propose that cell cycle remodeling is not a passive outcome but an active driver and a key barrier of reprogramming. The longer G1 phase in somatic cells, maintained by high levels of CKIs such as p21 and p16, constitutes a major obstacle because it provides a window for differentiation signals and activates tumor suppressor pathways under reprogramming pressure. Successful reprogramming requires overcoming these barriers by inhibiting the p53/p21 and p16/Rb pathways and by exogenous expression of core pluripotency factors to upregulate proliferative Cyclins and CDKs. This drives metabolic and epigenetic shifts toward a glycolytic state and open chromatin, further promoting cell cycle acceleration. The resulting shortened G1 phase limits exposure to differentiation signals and creates favorable conditions for establishing pluripotency. However, this forced rapid proliferation comes at the cost of increased genomic instability due to replication stress. Therefore, transiently modulating cell cycle checkpoints or strategies targeting specific cell cycle phases are crucial for improving the efficiency and quality of iPSC generation.

## 7 A practical toolkit for manipulating the cell cycle in stem cell biology and reprogramming

The intricate coupling between the cell cycle and cell fate presents both a challenge and an opportunity for experimental manipulation. This toolbox summarizes practical strategies for harnessing cell cycle regulators to improve the efficiency and quality of stem cell maintenance, differentiation, and reprogramming (Table 3). Key reagents, applications, and crucial considerations are outlined below.

**TABLE 3:** Summary of the experimental toolkit for cell cycle manipulation.

Strategy	Tool examples	Operation plan	Main applications	Key considerations
G1 phase synchronization to enhance stemness/reprogramming	Palbociclib (CDK4/6i)	Stem cell maintenance: long-term addition at low doses (100–500 nM). Reprogramming: add early in the process (days 0–4) and remove later.	Improve reprogramming efficiency; maintain stem cell quiescence	Effects are reversible; dose and duration must be optimized to avoid permanent arrest.
Managing replication Stress	AZD1775 (WEE1i), MK-8776 (CHK1i)	Research use: add at very low doses (nM range) in rapidly proliferating stem cells. Basic research: understand the relationship between replication stress and genomic stability.	Therapeutic window is extremely narrow; High doses cause DNA damage; use with caution in clinical settings.	Extremely narrow therapeutic window; high doses cause DNA damage; use with caution in clinical settings
Transient p53 inhibition to improve efficiency	Pifithrin-α (small molecule), shRNA-p53	Use only during the critical reprogramming window (e.g., days 2–8); must be completely removed after completion.	Improve iPSC yield from hard-to-transfect or senescent cells.	Safety core lies in transient inhibition; must validate final iPSC clones for p53 pathway function and genomic integrity.
Cell cycle phase-specific manipulation	FUCCI reporter system	1. Label cells with FUCCI; 2. Short G1 phase cells for reprogramming or differentiation; 3. Develop phase-specific promoters to drive key factors.	Maximize reprogramming/differentiation efficiency; Basic mechanism research.	Requires cell engineering; this is the most cutting-edge and precise strategy.

### 7.1 Modulating CDK4/6 and CDK2 activity: driving proliferation and fate decisions

1. Tools:
  • CDK4/6 inhibition: Palbociclib (PD-0332991), Abemaciclib, Ribociclib (highly selective ATP-competitive inhibitors).
  • CDK2 inhibition: CVT-313, SU9516.
2. Strategy:
  • Stem Cell Maintenance: Long-term, low-dose treatment (100–500 nM Palbociclib) can reversibly arrest cells in G1, reduce spontaneous differentiation, and promote the preservation of a quiescent stem cell pool. The inhibitor must be withdrawn for subsequent expansion or differentiation.
  • Enhancing Reprogramming: Adding a CDK4/6 or CDK2 inhibitor during the early phase (days 0–4) of somatic cell reprogramming synchronizes the population in G1, a state perceived to be more permissive for reprogramming initiation. Subsequent withdrawal in the middle/late phase, potentially combined with pro-proliferative signals (e.g., Vitamin C), can enhance clonal expansion of successfully reprogrammed cells, thereby significantly increasing iPSC yield (Rais et al., 2013).
  • Prospects and Notes: This approach is valuable for optimizing organoid cultures, iPSC generation, and the expansion of stem cells for therapeutic products. The well-established safety profiles of CDK4/6 inhibitors in oncology provide a valuable reference for translational applications.

### 7.2 Fine-tuning replication stress management *via* WEE1/CHK1

1. Tools:
  • WEE1 inhibition: AZD1775 (Adavosertib).
  • CHK1 inhibition: MK-8776 (SCH900776), LY2606368 (Prexasertib).
2. Strategy:
  • Alleviating Replication Stress: Low-dose (nM range, requires meticulous titration) inhibition of WEE1 or CHK1 in rapidly proliferating PSCs can promote replication fork progression and alleviate endogenous replication stress, thereby reducing DNA damage accumulation (Rogers et al., 2020; Saini et al., 2015).
  • Purging Low-Quality Cells: Transient, low-dose CHK1 inhibition can be applied after high-throughput screening to selectively eliminate subpopulations with high replication stress and DNA damage, thereby enriching for healthier cells.
  • Prospects and Notes: Primarily a research tool for investigating the role of replication stress in cell fate decisions. Clinical translation requires extreme caution due to the risk of genomic instability.

### 7.3 Transient p53 inhibition: safely enhancing reprogramming efficiency

1. Tools:
  • siRNA/shRNA: targeting *TP53* mRNA.
  • Small molecule inhibitors: Pifithrin-α (PFTα, soluble), Pifithrin-μ (PFTμ, mitochondrial p53 inhibitor).
2. Strategy and Safety Protocol:
  • Core Principle: Inhibition must be strictly transient. Apply inhibitors or induce shRNA expression only during the critical window of reprogramming (e.g., days 2–8).
  • Safety Imperative: The inhibitor must be completely withdrawn or shRNA expression shut off after reprogramming is complete (i.e., upon iPSC colony formation) to allow full restoration of the p53 pathway in the resulting iPSCs. This is non-negotiable for genomic fidelity.
  • Validation: iPSC clones must be rigorously validated for genomic integrity (karyotyping, genome sequencing) and functional p53 pathway response.
  • Prospects and Notes: This is a gold-standard method for reprogramming recalcitrant or senescent cells. Safety is entirely contingent on the transience of inhibition (Marión R. M. et al., 2009a).

### 7.4 FUCCI/CDK biosensors and timed factor delivery: Precision control

1. Tools:
  • FUCCI system: Lentivectors expressing mVenus-hGeminin (107-130)/mCherry-hCdt1 (30-120). Red (G1), Yellow (G1/S), Green (S/G2/M) (Sakaue-Sawano et al., 2008).
  • CDK activity sensors: CDK2 activity reporter (based on Cyclin E-AKAR FRET sensor).
  • Timed delivery: Inducible expression systems (e.g., tetracycline-inducible promoters), optogenetic tools for protein degradation (e.g., B-LID system).
2. Strategy:
  • Infect the target cell population with the FUCCI system to enable real-time, non-invasive monitoring and sorting of cells based on cell cycle phase *via* flow cytometry or live-cell imaging.
  • Experimental discovery: FACS-sorted G1 cells exhibit higher reprogramming competence.
  • Precision intervention: Engineer inducible expression systems activated in specific cell cycle phases (e.g., using a CDT1 promoter active in late G1) to drive the expression of critical reprogramming factors (e.g., GLIS1).
  • Prospects and Notes: It enables the dissection of cell cycle-specific events in fate decisions and paves the way for synchronized, high-quality, large-scale stem cell production protocols.

### 7.5 How to use this toolkit

1. Define the Goal: Determine if the aim is expansion, reprogramming, differentiation, or mechanistic study.
2. Select the Strategy: Choose the most appropriate approach from the table below.
3. Optimize *via* Pilot Studies: Perform dose- and time-response experiments to define optimal conditions for your specific cell type.
4. Rigorously Validate: Any intervention must be followed by functional assays to confirm the desired outcome (e.g., improved reprogramming efficiency, maintained pluripotency, successful differentiation) and, crucially, genomic stability.

Translating the theory of coupling between the cell cycle and pluripotency into practical applications requires a precise toolkit. This section provides a procedural guide for experimentally manipulating the cell cycle to achieve desirable outcomes in stem cell culture, reprogramming, and differentiation. We offer a toolbox that includes pharmacological inhibitors (such as CDK4/6 inhibitor Palbociclib, WEE1/CHK1 inhibitors, transient p53 inhibitors), genetic tools (siRNA/shRNA, inducible systems), and advanced biosensors (for example, FUCCI systems for real-time cell cycle phase tracking). The strategic use of these tools depends on the context: inhibiting CDK4/6 can be employed to synchronize cells in G1 to boost the initiation of reprogramming or to maintain stem cell quiescence. Transient p53 inhibition can bypass aging barriers in reprogramming but must be carefully controlled to avoid genomic instability. Additionally, real-time monitoring with FUCCI enables isolation of cells in specific cell cycle phases with diverse fate potentials. The overarching principle is the transient and precise nature of these interventions, aiming to mimic physiological transitions and to ensure that resultant stem cell populations are of high quality and safety for research and therapeutic applications.

## 8 Cell cycle machinery in ASCs

### 8.1 Cell fate decisions and cell cycle regulation in NSC

NSCs constitute a population capable of self-renewal and multipotent differentiation, capable of giving rise to neurons, astrocytes, and oligodendrocytes. They play a critical role in neural development, homeostasis, and repair following injury (Li X. Y. et al., 2022). The biological behavior of NSCs is tightly controlled by the cell cycle, and the precision of this regulation is essential for maintaining NSC stability and ensuring timely differentiation. In recent years, increasing evidence has shown that disruptions in the neural cell cycle regulatory network not only impairs normal neural development but are also closely associated with the occurrence of various neurodegenerative diseases and brain tumors.

In NSCs, the progression of the G1 phase is particularly critical, as it determines whether the cell continues proliferation or exits the cell cycle to undergo differentiation or remain quiescent. Similar to the process of somatic cell reprogramming, the complex of CyclinD and CDK4/6 phosphorylates Rb, relieving its inhibition of E2F transcription factors. This promotes the expression of *CyclinE* and activates CDK2, thereby facilitating the cell’s transition through the G1/S checkpoint into the S phase (Grison and Atanasoski, 2020). Rb family proteins inhibit the expression of cell cycle genes such as *CyclinA2/E2* and *CDK1/2* by suppressing the activation of E2F1/3, thus maintaining NSCs in a quiescent state. When Rb is absent, E2F is released from repression, leading to the expression of CyclinA2/E2-CDK1/2 complexes, promoting G1/S transition and triggering NSC activation and cell cycle progression (Fong et al., 2022). In NSCs, E2F3a and E2F3b function as cell cycle effectors that precisely regulate *Sox2* expression level, which counteract each other to modulate the transcription of the pluripotency factors. An overexpression of *Sox2* promotes progenitor self-renewal and S retention, whereas reduced Sox2 levels drive cell cycle exit and differentiation (Julian et al., 2013). Recent studies have shown that overexpression of *CDK4* shortens the G1 phase, while the loss of CDK6 results in decreased proliferative capacity of NSCs, suggesting that CDK6 may have a unique role in the cell cycle regulation of NSCs(Grison and Atanasoski, 2020; Shih et al., 2022). Additionally, research indicates that under hypoxic conditions, fetal NSC cell cycle progression is delayed, mainly characterized by G2/M phase arrest during the interphase nuclear migration process; short-term exposure to hyperoxia significantly shortens the cell cycle duration, whereas prolonged hyperoxia prolongs it. Both types of oxygen treatment promote the transition of cells from G0/G1 into the S or G2/M phases (Chou et al., 2022; Lanto et al., 2025). Notably, cell cycle regulation in NSCs exhibits clear spatiotemporal specificity, presenting distinct regulatory patterns across different brain regions and developmental stages. This heterogeneity may form a fundamental basis for the diversity within NSC populations.

In the adult mammalian brain, most NSCs remain in a quiescent state (Morales and Mira, 2019; Morshead et al., 1994), but specific external signals can trigger their exit from quiescence into a proliferative state—a dynamic transition termed reactivation. Among the regulatory factors, RingoA directly binds and activates CDK1/2, increasing the activation threshold of CDKs and promoting the re-entry of shallow quiescent NSCs into the cell cycle (Gonzalez et al., 2023). Moreover, Lrig1 facilitates cell cycle re-entry and EGFR response readiness by allowing an increase in *EGFR* levels while simultaneously limiting its signal activation (Marqués-Torrejón et al., 2021).

At the metabolic level, the mitochondrial pyruvate carrier (MPC), composed of heterodimers of MPC1 and MPC2, is an essential structural complex required for pyruvate transport (Herzig et al., 2012; Vanderperre et al., 2016). Studies have shown that *Mpc1* expression is highest in quiescent NSCs and gradually downregulated during differentiation. MPC-mediated mitochondrial import of pyruvate is a key fundamental for maintaining the metabolic homeostasis of the quiescent state (Petrelli et al., 2023). The core of NSC fate decisions lies in a dual regulatory mechanism of the cell cycle, with recent research revealing key pathways involved in its dynamic balance. In physiological quiescence, the Notch-Hey1 pathway continuously suppresses neuronal differentiation genes, collaborating with G1 phase arrest to sustain stemness. Meanwhile, Cend1 promotes cell cycle exit by blocking Notch signaling and remodeling the G1/S transition checkpoint (Harada et al., 2021; Wang R. et al., 2022; Zhang et al., 2020). Notably, both mechanisms ultimately converge at the G1/S transition hub. Furthermore, SUMOylation also plays a crucial role by modifying the Wts kinase to inhibit Hippo pathway activity, thereby relieving its repression of *Yki* and promoting NSC exit from quiescence into the cell cycle. This process is driven by amino acid-activated Akt signaling and provides a key molecular switch for neurogenesis and brain development in *Drosophila* (Gao et al., 2024).

In addition to positive regulators, cell cycle inhibitory proteins are equally important in NSC cycle regulation. CDK inhibitors such as p21 and p27 can bind to and inhibit CDK-Cyclin complexes, inducing NSCs to exit the cell cycle (Coqueret, 2003). Research indicates that p21 is expressed in adult NSCs and early neural progenitors, maintaining stem cell quiescence by inhibiting cell cycle progression; its absence leads to excessive proliferation and accelerated differentiation of NSCs(Chiani et al., 2024; Maeda et al., 2023; Pechnick et al., 2008). Foxg1 promotes neural progenitor cell proliferation by suppressing p21 and facilitates cell cycle exit and differentiation through the extension of the G1 phase, thereby coordinating the amplification and differentiation of neural progenitor cells (Wang J. et al., 2022).

Recent studies have revealed that non-coding RNAs, particularly microRNAs, play finely tuned roles in NSC cell cycle regulation. For instance, miR-199a-5p, miR-9, and miR-103-3p target key regulators of the Wnt/β-catenin pathway, such as GSK-3β, Hes1, and Ndel1, mediating G1/S transition *via* CyclinD1 and bidirectionally regulating the balance of NSC proliferation and differentiation (Li et al., 2022b; Yang Y. et al., 2023; Yuan et al., 2021). MiR-9-5p modulates self-renewal and differentiation by targeting the transcription factor Onecut2 (OC2) (Sharma et al., 2024). lncRNAs, such as Pnky, have been found to regulate NSC cell cycle exit and neuronal differentiation through interaction with PTBP1(Ramos et al., 2015). *In vitro* hypoxia/reoxygenation (OGD/R) induced NSCs, the lncRNA H19 alleviate p53-mediated G1/S checkpoint arrest by inhibiting p53 transcriptional activity and expression levels, thereby promoting cell proliferation and differentiation after OGD/R (Gan et al., 2022).

Aberrant regulation of NSC cell cycle progression is closely associated with the occurrence and progression of various neurological diseases. In neurodegenerative disorders such as Alzheimer’s disease, endoplasmic reticulum (ER) stress activates the Ire1/Xbp1 pathway, which suppresses the transcription of *E2F1*, leading to G1/S cell cycle arrest in NSCs. This impedes their normal proliferation and induces apoptosis, thereby damaging neural homeostasis (Aceves et al., 2024). Abnormal activation of the p16-Rb pathway is considered a significant factor contributing to decreased neurogenesis in aged individuals. Gene manipulation that inhibits *p16* expression can partially restore neurogenic capacity in aged mice (Si et al., 2021). Studies have also demonstrated that CDK4/6 inhibitors such as Palbociclib and N1J effectively suppress the proliferation of glioblastoma stem cells (GSCs), showing promising clinical prospects for glioblastoma treatment (Li M. et al., 2017; Sun et al., 2025). However, the presence of NSC-like tumor cells renders CDK4/6 inhibitors ineffective in significantly improving the survival of glioblastoma patients. Recent studies have revealed that tGLI1 is a key driver of multi-drug resistance in glioblastoma. The FDA-approved drug KCZ targeting tGLI1, in combination with CDK4/6 inhibitors, has demonstrated synergistic antitumor effects in preclinical models, offering a mechanism-dependent combination therapeutic strategy for glioblastoma (Yu et al., 2025).

In NSCs, the cell cycle does not merely govern cell proliferation. It is a central hub that integrates internal and external signals to determine the balance among quiescence, activation, and differentiation. This section discusses how cell cycle progression, particularly the G1/S transition, governs NSC fate within neurogenic niches. Quiescent NSCs are maintained in a reversible G0 state by high levels of CKIs such as p21 and p27. Activation to enter the cell cycle is a tightly regulated process involving downregulation of CKIs, upregulation of D-type Cyclins, and participation of specific signals such as RingoA and LRIG1. Metabolic status, especially mitochondrial pyruvate input mediated by MPC, is closely linked to maintenance of quiescence. Additionally, this section summarizes the role of noncoding RNAs in fine-tuning the expression of cell cycle regulators and key signaling pathways (Notch, Wnt/β-catenin). Disruptions in this precise control, such as persistent endoplasmic reticulum stress or abnormal expression of *p16*, can lead to age-related neurogenesis decline and neuropathologies (including gliomas), for which cell cycle targets offer therapeutic prospects. Therefore, in NSCs, the cell cycle is a key determinant of their regenerative potential and homeostatic function.

### 8.2 Commonalities and specificities of cell cycle regulation in ASCs

HSCs are a typical cell type in a deeply quiescent state. Studies have shown that p57 is a key molecule maintaining HSC quiescence, and its loss leads to accelerated cell cycle progression and exhaustion of HSCs(Matsumoto et al., 2011). p21 and p27 also fulfill essential regulatory roles. Their expression is upregulated following external stressors, including DNA damage, thereby contributing to the maintenance of HSC quiescence and homeostasis (Cheng et al., 2000). This “triple safety net” ensures that HSCs are activated only in response to strong regenerative signals. Intestinal stem cells (ISCs) are classic quiescent reserve stem cells. They also express high levels of *p57* and *p21* to sustain quiescence. Upon injury, these quiescent ISCs can be rapidly activated to replenish the rapidly cycling Lgr5+ ISCs(Buczacki et al., 2013). This indicates that even in a rapidly renewing tissue, a quiescent stem cell protected by CKIs is required as a backup. Muscle satellite cells (MuSCs) at steady state are almost entirely quiescent. p27 and p21 are central to maintaining their quiescence. Studies show that MuSCs lacking p27 spontaneously enter the cell cycle and eventually exhaust due to excessive proliferation, losing regenerative capacity (Chakkalakal et al., 2012). Thus, CDKIs are a common key group of proteins maintaining quiescence across multiple ASCs. Their function is to establish a high cell-cycle threshold to prevent unnecessary proliferation in the absence of proper cues, thereby preventing premature cellular senescence.

Despite the universal regulation by CDKIs, signals from distinct niches are also organized in a tissue-specific manner to tailor these cell-cycle components to their unique physiological functions. In HSCs, TGF-β strongly inhibits cell-cycle progression by upregulating CKIs such as p57 and p21 (Yamazaki et al., 2009). Angpt1 promotes adhesion of HSCs to the bone marrow niche *via* its receptor Tie2 and likewise maintains quiescence by upregulating *p27* (Arai et al., 2004). Therefore, HSCs primarily maintain quiescence by restricting entry into the cell cycle. In contrast, Lgr5+ ISCs at the crypt base reside in a microenvironment with high Wnt signaling. The core downstream effector of Wnt signaling, β-catenin, directly transcriptionally activates proto-oncogenes such as *Cyclin D1* and *c-Myc*, driving G1/S transition (van der Flier and Clevers, 2009). Consequently, ISCs primarily promote cell-cycle progression to sustain rapid renewal of the intestinal epithelium. Quiescent ISCs reside in a region with a lower Wnt signaling gradient at the upper part of the crypt. MuSC quiescence is largely maintained by physical adhesion to muscle fibers and by its specialized basement membrane. Notch signaling plays a major role in maintaining MuSC quiescence. Upon injury or after exercise, inhibition of TGF-β family members is relieved, while mitogens such as FGF and HGF are released. These signals act synergistically to downregulate CKIs such as p27 and upregulate *Cyclins/CDKs*, driving MuSC activation and entry into the cell cycle for repair (Yin et al., 2013). Distinct types of ASCs precisely modulate the activity of core cell cycle proteins through unique signaling configurations to adapt to the physiological demands of their respective niches.

### 8.3 HSC quiescence control: mechanisms and transplantation applications

HSCs are a well-established model for research on cell cycle quiescence and the maintenance of stemness, both of which have important clinical implications. The functional homeostasis of HSCs depends on the preservation of their quiescent state, the precise regulation of signaling pathways, and the dynamic balance of the cell cycle. Elucidating the core molecular mechanisms not only deepens our understanding of hematopoiesis but also provides critical targets for optimizing hematopoietic stem cell transplantation (HSCT) strategies.

The quiescent state of HSCs (G0 phase) is a key safeguard of their long-term self-renewal capacity. This state is finely regulated by CKIs. p21 directly inhibits the association of cyclin–kinase complexes by forming regulatory networks with molecules such as Dkk1, NF-Y, and Skp2, thereby hindering the G0-to-G1 transition. p57 augments the quiescence of HSCs by associating with cell-cycle regulators such as p27 and Cyclin D2. p18 similarly participates in maintaining quiescence through a comparable mechanism. Collectively, these CKIs constitute a regulatory network that prevents HSC exhaustion due to excessive proliferation. In addition, the quiescent state of HSCs is regulated through multiple signals from the bone marrow niche. For example, the TGF-β pathway inhibits proliferation by downregulating *c-Kit* and *IL6R* expression while also enhancing the activity of p21 and p57. Furthermore, Angiopoietin-1 (Angpt1) binds to its receptor Tie2, which strengthens adhesion between HSCs and osteolineage cells in the niche. This interaction helps anchor HSCs within a microenvironment that supports low proliferation. Notably, Tie2^+^ HSCs have been confirmed to be an Long-Term Self-Renewing population at the apex of the hematopoietic hierarchy (Mann et al., 2022).

Activation of HSCs from quiescence and entry into the cell cycle is a multi-signal-coordinated process in which the c-Kit signaling axis plays a pivotal “on/off” role. c-Kit, a type III receptor tyrosine kinase, dimerizes upon binding its ligand stem cell factor (SCF) and undergoes autophosphorylation of intracellular tyrosine residues. In particular, the juxtamembrane region (JM) contains core phosphorylation sites Y568 and Y570: Y568 recruits signaling molecules such as Src family kinases (SFK) and SHP-2, while Y570 mediates binding of SHP-1 and Shc, thereby initiating downstream signaling. However, c-Kit–mediated proliferative signaling is constrained by multiple negative regulators, including SHP-1 (binding Y570) and SHP-2 (binding Y568), which, as protein tyrosine phosphatases (PTPs), can dephosphorylate key tyrosine residues on c-Kit to inhibit activation of pro-proliferative pathways such as PI3K/MAPK, creating a “signal brake” mechanism (Raghav and Gangenahalli, 2018). The small-molecule compound NSC87877 can specifically inhibit the enzymatic activities of SHP-1 (IC50 = 0.355 μM) and SHP-2 (IC50 = 0.318 μM), thereby relieving their inhibition of c-Kit signaling and cooperatively promoting proliferation with SCF(Raghav et al., 2018a). In K562 cells sorted in the G0/G1 phases, pre-treatment with NSC87877 followed by SCF addition (Pre-treatment) increased the proliferation rate to 40.28%, markedly higher than the control (29.25%) and SCF-alone treatment (33.27%) groups (Raghav et al., 2018b). In the human megakaryoblastic cell line MO7e, this pre-treatment raised the S-phase cell fraction from 17.89% (control) to 53.88%, and the proportion of Ki-67^+^ cells also rose significantly, indicating that the synergistic action of NSC87877 and SCF effectively promotes the transition of HSCs from quiescence to the proliferative cycle (Raghav et al., 2018a).

Activation of the c-Kit signaling pathway triggers deeper metabolic and signaling rewiring. Resting HSCs rely on FoxO3a-mediated transcriptional control to maintain high expression of antioxidant enzymes, ensuring a low ROS environment that sustains self-renewal and quiescence (Miyamoto et al., 2007). Following SCF binding to c-Kit, PI3K/Akt signaling is activated, inhibiting FoxO3 function and steering the metabolic program toward mitochondrial OXPHOS to supply energy for proliferation (Liang and Ghaffari, 2017; Mann et al., 2022). Concurrently, the MAPK pathway is co-activated, downregulating cell cycle inhibitors such as p21, thereby relieving cell cycle arrest and collectively driving the transition of HSCs from quiescence to proliferation (Mann et al., 2022; Raghav and Gangenahalli, 2018).

Notably, although SCF treatment reduces surface c-Kit expression, inhibition of SHP-1/SHP-2 with NSC87877 markedly increases phosphorylation at key c-Kit tyrosine residues (e.g., pY568/pY570), indicating that the activation state of c-Kit, rather than its expression level, is the critical regulator of HSC proliferation. This finding explains why low-dose SCF (20 ng/mL) in combination with NSC87877 (5 μM) robustly promotes proliferation, reducing reliance on high-dose SCF in clinical applications (Raghav et al., 2018a; Raghav et al., 2018b).

The above molecular mechanisms provide multiple potential targets to optimize HSCT strategies. For example, improved HSC quiescence can be achieved by inhibiting Dkk1, whereas activating Angpt1/Tie2 signaling can enhance engraftment efficiency post-transplantation. The synergistic activation strategy of the c-Kit pathway, particularly the NSC87877 and SCF combination, has become a core approach for *ex vivo* HSC expansion. Studies show that pre-treating bone marrow–derived CD34^+^ HSCs with NSC87877 for 1 h before adding SCF increases proliferation by approximately 50%, and significantly enhances clonogenic capacity while preserving multipotency (Raghav and Gangenahalli, 2018; Raghav et al., 2018a). This strategy can also reduce SCF usage from 80 ng/mL to 20 ng/mL, lowering clinical costs and minimizing potential side effects (Raghav et al., 2018a; Raghav et al., 2018b).

In summary, quiescence maintenance and activation-driven proliferation of HSCs are governed by multi-layer regulation, including the CKI network, multiple signaling pathways (TGF-β, Angpt1–Tie2, c-Kit), and metabolic reprogramming. Among these, the cooperative activation of c-Kit signaling by SCF and NSC87877, by counteracting the negative regulation of SHP-1/SHP-2, provides an effective strategy for *ex vivo* expansion and maintenance of HSC stemness in HSCT. Further optimization of pretreatment regimens is expected to significantly enhance both the quantity and quality of HSCs required for transplantation, ultimately improving the clinical outcomes of HSCT (Raghav and Gangenahalli, 2018; Raghav et al., 2018a; Raghav et al., 2018b).

The regulation of quiescence and proliferation in ASCs is a highly tailored process that is essential for long-term tissue maintenance and regenerative capacity. This section compares the cell cycle control mechanisms across different types of ASCs, highlighting their general dependence on CKIs such as p21, p27, and p57 to enforce a deep quiescent state (G0) and prevent exhaustion. However, responses to niche signals that trigger activation show significant tissue specificity. HSCs predominantly respond to signals that reinforce quiescence, with activation involving precise coordination through receptors such as c-Kit and their negative regulators (SHP-1/SHP-2). In contrast, ISCs exist in a high-Wnt environment, which, *via* expression of Cyclin D1, actively promotes proliferation while maintaining quiescent reserve stem cells as a backup. MuSCs represent another pattern, in which the quiescent phase is maintained by inhibitory signals and is disrupted by pro-activation signals after injury. This comparative analysis indicates that core cell cycle machinery is broadly conserved but is finely tuned by niche-specific signaling networks to meet each tissue’s unique homeostasis and regenerative needs.

## 9 Conclusion

In recent years, with the continuous advancement of stem cell research, the coupling mechanisms between stemness maintenance and cell cycle regulation have become a focal point in life sciences. Multiple studies confirm that the unique cell cycle characteristics of stem cells, particularly their rapid proliferation ability, are fundamental to maintaining pluripotency and self-renewal. This process is regulated not only by classical cell cycle regulators such as Cyclins and CDKs, but also involves complex regulatory networks with pluripotency-associated transcription factors. The interaction between cell cycle proteins and pluripotency factors in stem cells not only modulates cell cycle progression but also directly influences the expression levels of pluripotency genes, establishing a robust link between proliferation and the maintenance of stemness. Meanwhile, important cell cycle inhibitory proteins such as p21 and p27 play significant roles in regulating self-renewal, ensuring that stem cells proliferate rapidly while avoiding genomic instability by controlling cell cycle entry and arrest.

Regarding regulatory mechanisms, the unique cell cycle structure of ESCs results from the coordinated action of multiple layers of regulation, including epigenetic modifications, signaling pathways, metabolism, and cell cycle regulators (Nardiello et al., 2011). Epigenetic modifications play an indispensable role in ESC cell cycle regulation; chromatin remodeling and changes in its modification status regulate the expression of cell cycle genes. For example, the cooperation between DNA demethylase Tet1 and PRC2 enhances H3K27me3 marks, stabilizing the expression of pluripotency genes (Chrysanthou et al., 2022a; Ficz et al., 2011). Moreover, signaling pathways provide external regulatory cues that support the cell cycle architecture of ESCs. Pathways such as Jak/Stat3, MAPK/Erk, and PI3K/Akt play dual roles in maintaining pluripotency and regulating the cell cycle (Singh et al., 2014).

On the one hand, these mechanisms promote rapid cell proliferation; on the other hand, they activate mitotic regulators, integrating environmental signals with intracellular cell cycle regulation. Furthermore, alterations in glycolytic metabolism are intimately linked to cell cycle control. Elevated glycolytic activity not only supplies rapid energy for stem cells but also regulates the expression of cell cycle proteins, thereby facilitating stem cell proliferation and the maintenance of pluripotency (Dong et al., 2024). This network—the interplay among metabolism, the cell cycle, and stemness—offers a novel perspective for understanding stem cell self-renewal and presents potential targets for metabolic regulation of stem cell states.

Looking ahead, there remains significant potential for research into the coupling mechanisms between stem cells and the cell cycle. Our preliminary studies utilizing CRISPR/Cas9 systems have explored precise modulation of specific regulatory factors to achieve controllable stem cell states (Wang C. C. et al., 2022). As high-throughput sequencing, single-cell analysis, and gene editing technologies continue to advance, it is anticipated that more interactions between cell cycle regulators and pluripotency factors will be uncovered, including their differential regulatory mechanisms across various stem cell types and developmental stages. Future research directions also include the synchronized regulation of cell cycle dynamics and chromatin structural changes. Specifically, how to ensure rapid stem cell proliferation while maximizing genomic stability and preserving genetic integrity. From a clinical perspective, understanding the intricacies of cell cycle regulation in stem cells may help optimize *in vitro* culture systems, thereby enhancing the safety and efficacy of regenerative medicine therapies.

In addition, strengthening research on the mechanisms of stem cell tumorigenesis should be regarded as a key future direction. Because rapidly proliferating stem cells share characteristics with tumor cells, investigating the relationship between cell cycle dysregulation and tumor development holds potential significance for cancer prevention and treatment. Recently, a research team in Canada examined how cell cycle duration influences tumor susceptibility, discovering that in various tumor types, the overall cell cycle length can predict susceptibility to malignant transformation (Chen D. N. et al., 2025). Future studies should also focus on developing novel strategies that ensure efficient cell proliferation while effectively preventing genomic instability and tumorigenesis.

Cell cycle regulators play a dual role in stem cell fate determination and safety. Traditional stem cell expansion strategies often emphasize proliferative signals while neglecting the genomic instability and tumorigenic risk arising from cell cycle abnormalities. In recent years, cell-cycle–targeted drugs, exemplified by CDK4/6 inhibitors, have provided new approaches to maintaining stemness while preserving genomic integrity and safety. Using CDK4/6 inhibitors (such as Palbociclib, Abemaciclib) can artificially arrest stem cells in the G0/G1 phase, forcing them into a quiescent state. Theoretically, this can prevent exhaustion due to excessive proliferation and sustain long-term self-renewal. For example, in HSCs, high expression of CIP/KIP family CDK inhibitors such as p57 and p27 is central to maintaining quiescence. Studies show that CDK6 loss or pharmacological inhibition can promote HSC quiescence, reduce differentiation, and thereby protect stem cells from chemotherapy-induced damage (Laurenti et al., 2015). This inhibitory effect is typically reversible. Upon drug withdrawal, the cell cycle arrest is lifted and stem cells can re-enter the cycle to proliferate and differentiate. This reversibility is one of the greatest advantages for stem cell culture applications. Researchers can switch flexibly between conditions that require expansion (no inhibitor) and those that require maintenance of quiescence or protection from damage (with inhibitors). For instance, in hPSC culture, brief, low-dose Palbociclib application effectively suppresses spontaneous differentiation and maintains the expression of pluripotency markers, and after withdrawal, normal proliferation and differentiation capabilities can be restored without observed permanent cell-cycle arrest (Pauklin and Vallier, 2013). It is important to note that this forced quiescence acts as a double-edged sword. While it can preserve stem cell function, excessive or irreversible inhibition may prevent stem cells from responding to normal regenerative signals. This could result in functional exhaustion and impaired tissue repair.

In clinical-stage stem cell applications, manipulating the cell cycle can optimize proliferation and differentiation strategies to reduce tumorigenic risk. One approach is to rapidly expand cells using pro-proliferation signals, followed by a short introduction of CDK4/6 inhibitors or other cell cycle blockers such as mTOR inhibitors to induce a “pause” and allow DNA repair, thereby increasing genomic integrity during expansion. This dynamic regulation more closely mimics the cyclic control of *in vivo* stem cells and is advantageous for maintaining a healthy stem cell compartments compared with continuous stimulation or inhibition. Additionally, residual undifferentiated hPSCs pose a major tumorigenic risk due to their limitless proliferative potential. Targeting cell cycle checkpoints to eliminate these cells is a highly promising strategy. Antiproliferative agents such as 5-fluorouracil (5-FU) can selectively kill rapidly proliferating undifferentiated hPSCs while having relatively little impact on differentiated cells that have slowed or exited the cell cycle.

Our review synthesizes prior work in the field and proposes that the cell cycle is not merely the central driver of cell division but also a key component of stemness maintenance and fate determination. The coupling between a shortened cell cycle (especially a short G1 phase) and pluripotent states is mediated by a multi-faceted network, including reciprocal regulation between core pluripotency factors and Cyclin-CDK complexes, integration of key signaling pathways, dynamic epigenetic remodeling, and metabolic reprogramming. This interconnected system ensures tight coupling of rapid proliferation with differentiation suppression. Looking forward, to dissect causality in depth, advanced single-cell technologies and real-time biosensors should be employed to resolve the temporal sequence of these interactions during fate transitions. A major translational challenge lies in how to harness this knowledge to safely and effectively manipulate the cell cycle. For example, by using transient CDK inhibition to enhance reprogramming efficiency or expand clinically relevant stem cells without compromising genomic integrity. Moreover, studying how cell cycle dysregulation contributes to stem cell–derived pathologies (such as teratoma formation) is crucial for developing safer regenerative therapies. Cracking the precise regulation of the cell cycle will accelerate the translation of stem cell research into medical and biological applications.
